# What drives participation in community-based forest management? Insights from a global review

**DOI:** 10.1007/s13280-025-02278-7

**Published:** 2025-11-27

**Authors:** Prabin Bhusal, Rajan Parajuli, Erin O. Sills

**Affiliations:** 1 Department of Forestry and Environmental Resources, North Carolina State University, Raleigh, NC 27695 USA; 2 Institute of Forestry, Tribhuvan University, Pokhara Campus, Hariyokharka-15, Pokhara, Nepal

**Keywords:** Effect size, Forest governance, Meta-analysis, Participatory forest management, Publication bias

## Abstract

Community-based forest management (CBFM) is widely promoted as a strategy that links forest management with local livelihoods through participatory governance. This global review used novel systematic review methods to evaluate predictors of people’s participation in CBFM. Based on 66 cases from 47 studies across 18 countries, we identified 248 predictors that have been used to explain people's participation in CBFM and categorized them into seven broad categories. While demographics, household size, and landholding size are the most frequently tested, factors such as off-farm household income, leadership style, and forest condition are less commonly tested yet more often statistically significantly related to participation in CBFM. The meta-regression revealed that the specific type of CBFM (the institutional model) moderates the effects of certain predictors. These results highlight the multifaceted and context-specific drivers of participation in CBFM, underscoring the need for both household- and community-level strategies to foster effective forest governance.

**Supplementary Information:**

The online version contains supplementary material available at 10.1007/s13280-025-02278-7.

## Introduction

The socio-economic and ecological significance of local people's participation in forest management is widely recognized (Agrawal and Gupta 2005; Oldekop et al. 2018; Hajjar et al. 2021). Community-based forest management (CBFM) is a decentralized approach that centers on local participation in forest management and decision-making, allowing them to utilize and manage forests (Tole 2010; Smith et al. 2023). Hajjar et al. (2021) reported that 14% of the global forests and 28% of forests in countries with low- and middle-income are governed by CBFM. CBFM has gained global recognition as a remedy for deforestation and forest degradation (Oldekop et al. 2019) which generates both socio-economic and ecological benefits (Gilmour 2016; Erbaugh et al. 2020). Recognition of this governance approach has transformed local communities from resource degradation agents to partners in sustainable forest management and restoration (Agrawal et al. 2023).

Different models of CBFM have been implemented worldwide, including community forestry, participatory forestry, joint forest management, collaborative forest management, participatory forest management, and village forests. Across all these models, one key issue is the extent of people’s participation. This varies across the different models of CBFM, with relatively less participation in conservation-focused regimes and relatively more participation in regimes with a bottom-up approach to defining management activities and access to resources (Gilmour 2016). For example, Nepal's community forestry program, launched in the early 1990s, grants local communities the right to use, access, share benefits, and manage forest resources by establishing of community forest user groups (Gautam et al. 2023). Similarly, Cameroon's 1994 Forest Law transferred forest management rights to local people through forest councils, enabling them to use and benefit from forest resources (Kimengsi and Deodatus Ngu 2022). In Ethiopia, participatory forest management allows local communities to utilize forest products, while also taking responsibility for conserving forests from damage and encroachment (Bakala et al. 2021). While these three cases allow for substantive local participation, actual participation varies across systems, communities, and households.

Literature has extensively documented the importance of people's participation in CBFM, emphasizing its pivotal role in fostering collective action, planning and decision-making, and ensuring sustainable resource management and good governance (Padgee et al. 2006; Chhetri et al. 2013; Gautam et al. 2023; Bhandari et al. 2018). Participation in CBFM fosters a sense of ownership and responsibility among local communities (Pokharel and Tiwari 2013), contributing to establishing trust and reciprocity (Kimengsi and Bhusal 2021). It promotes social cohesion and empowerment by providing marginalized groups with a voice in forest governance and management, thereby enhancing inclusive and equitable decision-making processes (Bhandari et al. 2018). Chaudhary et al. (2016) and Poudel et al. (2018) found that involving local users in activities such as forest patrols, fire control, and monitoring illegal harvesting led to significant improvements in forest health and other ecological outcomes. Conversely, lack of local participation in forest management has been found to result in forest loss and degradation (Ojha and Hall 2021).

Similarly, past systematic reviews concluded that participation is a key factor in success of CBFM. Padgee et al. (2006) examined 31 articles on community forestry, including 69 case studies from around the world, to identify variables that have a significant effect on the success of community forestry underscoring participation as one of the key factors for the success of community forestry across the globe. Shyamsundar and Ghate (2014) discussed the community willingness to manage forests as a key condition for the success of community forestry. Similarly, Baynes et al. (2015) identified participation as a positive factor for the success of community forestry groups. Hajjar et al. (2021) conducted a global review of community forests focusing on their social and environmental effects and concluded that there are strong positive socio-environmental outcomes of people's participation in CBFM. Additionally, past studies have called for inclusiveness, equity, transparency, and accountability in decision-making processes to promote and facilitate local participation (Agrawal and Gupta 2005; Lamichhane and Parajuli 2014; Negi et al. 2018). Likewise, forest policies in many countries incentivize participation as the foundation for the sustainable development and management of community forestry (Agrawal and Gupta 2005; Kimengsi and Ngu 2022).

While there are numerous case studies of the factors influencing participation in CBFM in scientific literature, there has been no global synthesis of this literature. For instance, Bista et al. (2023) identified migration, caste, and access to training as key factors explaining the active involvement of local people in community forestry in Nepal, whereas Apipoonyanon et al. (2000) highlighted family size, knowledge, and institutional benefits as determining factors of people’s participation in Thailand. Most of these studies were based on household surveys covering a limited geographic area and a single point in time. We contribute to the literature and CBFM practices globally by conducting a systematic review of the factors that explain people’s participation in CBFM, considering methodological factors as well as characteristics of the institutions, study sites, and households, and carefully testing for publication bias. We aimed to identify the predictors that have been most frequently examined (most common) and most often found to be statistically significant (most important) in explaining people's participation in CBFM, as well as factors that have not been sufficiently explored.

Another important contribution of this study is the application of novel systematic review methods and tools to trace and predict people’s participation in CBFM globally. We employ both qualitative assessments and quantitative statistical techniques to draw insights on the factors influencing people's participation in CBFM. Specifically, we explored: (i) the most common and important predictors of participation, (ii) the strength and direction of the relationships between these predictors and participation, (iii) potential publication bias at the study level and for consistently tested predictors; and (iv) the influence of contextual and methodological factors affecting the relationship between the most common predictors and people's participation in CBFM. This global synthesis can help forest managers and policymakers better understand the drivers of participation in CBFM and identify key elements that foster effective community engagement across diverse socio-ecological systems.

## Methodology

### Article search, selection, and inclusion strategy

We implement a systematic review and meta-analysis of publications related to people’s participation in CBFM worldwide, following PRISMA (2020) guideline for systematic reviews (e.g., Liberati et al. 2009; Frey 2023) and Pigott and Polanin’s (2019) recommendations. To identify relevant studies, we employed a systematic search for peer-reviewed, English language publications from 2000 to 2023 that used quantitative statistical methods to analyze predictors of participation in CBFM globally (Supplementary Material Table S1 and Table S3). Using Google Scholar and Web of Science, we searched for articles using keywords and search terms^1^ that aligned with the study objectives and inclusion criteria, including participation level, willingness, engagement, and participation behavior. During the initial identification phase, we reviewed article titles, years of publication, abstracts, and statistical analyses to eliminate irrelevant articles. We retained articles that met our inclusion criteria, removed redundant ones, and uploaded their complete copy to Mendeley (Mendeley n.d.). A total of 104 articles were collected from the initial round of identification.

**Fig. 1:**
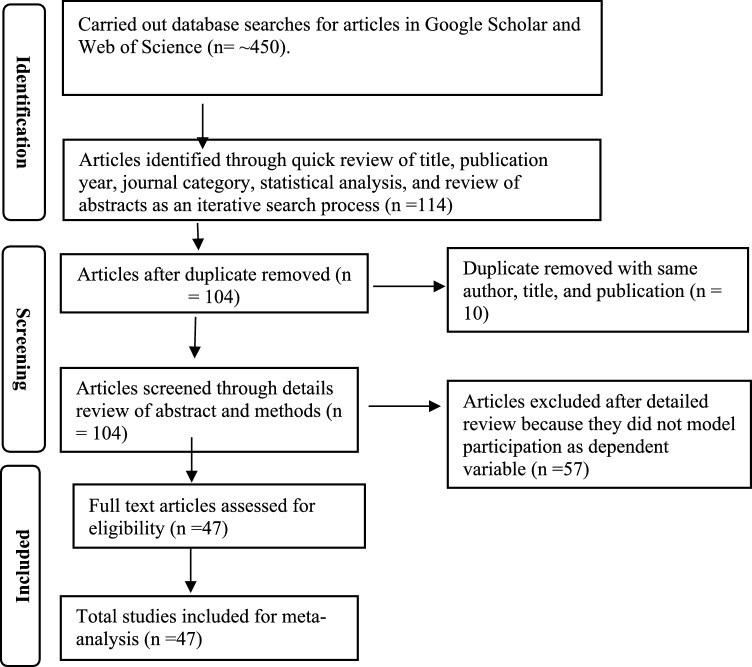
Flow diagram for studies included in a systematic review

We further examined the 104 selected articles from the initial screening and carefully reviewed the abstract to confirm that the article employed statistical methods to model participation in CBFM practices (Fig. 1). Following Pigott and Polanin (2019)’s best practices for abstract screening, we retained 47 articles that clearly met our inclusion criteria (Supplementary Material, Tables S1 and S3). We conducted a full-text review of these articles to fully code their estimation results for our vote counting analysis.

### Constructing the database, categorization and coding

We developed a coding framework to systematically categorize and analyze the independent variables in the 47 included articles. Table S2 presents a detailed description of characteristics of each study that we extracted from our coding, including the various participation factors and their reported associations with participation (positive, negative, or insignificant). While all the studies modeled people's participation in CBFM as the dependent variable, they can be divided into studies that examined ex ante willingness to participate in CBFM ("stated participation") and studies that examined current or past involvement in CBFM ("revealed participation"). Similarly, independent variables were iteratively grouped into seven broader categories based on author definitions and established literature. For instance, in the "Theory of Planned Behavior" category, we implemented the methodology described by Apipoonyanon et al. (2020) in their respective articles, which outlined the predictors in each category. In some cases, categories were not explicitly mentioned; therefore, we resorted to information available in the literature and our first-hand knowledge of community forestry.

We systematically categorized all independent variables modeled in the literature into seven categories (see Supplementary Material Table S2 for detailed descriptions) as: (I) the theory of planned behavior category describing the behavioral intentions and beliefs about participation and attitudes about the importance of community forestry; (II) the benefits from the participation category including material and socio-institutional gains from CBFM; (III) the biophysical category including forest attributes and other infrastructures; (IV) the economic category including all the economic attributes of forests and forest users used to explain people’s participation; (V) the governance category incorporating decision-making, policy implementation and community engagement; (VI) the institutional arrangement category encompassing resource access and the forest management institution (community or participatory); and (VII) the social category incorporating demographics, social structures, forest dependency, perceptions toward forest management, and social dynamics.

We thoroughly checked the database entries for each article, verifying the statistical significance and direction of the association of each predictor. In addition, we transcribed each predictor's description as stated in the publications. For instance, Bista et al. (2023) described age as the age of the household head, while Savari et al. (2020) defined it as the age of the respondent. This meticulous approach of data management and validation ensured the robustness and reliability of the information utilized in our meta-analysis.

### Meta-analysis of prevalence (proportional meta-analysis) to estimate predictor strength and analyze publication bias

Meta-analysis combines the results of multiple studies to provide a weighted average estimate (Barendregt et al. 2013; Barker et al. 2021). In this study, we used proportional meta-analysis to assess how often predictors of participation in CBFM were statistically significant both within individual studies and across studies based on methods outlined by Barker et al. (2021) and Barendregt et al. (2013). We conducted a publication bias test to determine whether the predictors are, in fact, consistently statistically significant and important for describing people's participation in CBFM or if they may only appear to be statistically significant due to publication bias, including only when they are statistically significant.

We estimated the measure of strength and consistency of predictors for each study case at the study level by assessing the proportion of significant predictors of people's participation (total significant events) across all tested independent variables (total modeled independent variables). Similarly, for each consistently tested predictor (tested ≥ 7, calculated from all study cases),^2^ we assessed the proportion of total significant events across the total number of times that each predictor was tested.

To account for methodological and contextual variation across studies, we employed random-effects models (Borenstein et al. 2009), applying the inverse variance method (Barendregt et al. 2013). We used the Freeman-Tukey double arcsine transformation of study-level proportions, but no transformation was used for the predictor-level analysis to stabilize the variance (Barendregt et al. 2013). We conducted a transformation for the study-level analysis because there were a few studies with variance at the extreme ends of the zero or one range, as discussed by Barker et al. (2021).

The forest plots were generated to show individual study or predictor-level estimates with 95% confidence intervals and pooled proportions, along with heterogeneity statistics. Similarly, we constructed a funnel plot and implemented the Egger's funnel plot symmetry test to examine for publication bias. The funnel plots are scatter plots that exhibit each study's effect size^3^ on the x-axis against its standard error on the y-axis. The Egger test provides a quantitative measure of publication bias (Egger et al. 1997; Lin et al. 2018) and shows the symmetry of the plot indicating unbiasedness. We conducted the analysis using R version 4.4.2 (2024) and the metaprop package (Viechtbauer 2010).

### Meta-regression of consistently tested predictors

By examining the distribution of t-statistics in the studies, we aimed to understand the sources of the heterogeneity in the results. We estimated meta-regressions to examine how t-statistics relate to study characteristics, including both the context and methodological factors (Atmadja and Sills 2015). We focused on commonly tested predictors: distance to forest, household head age, household size, land size, respondent age, respondent education, and respondent gender. The literature contains at least 30 coefficient estimates for each predictor except for respondent age. We calculated the t-statistics based on the coefficient and standard error when they were not explicitly reported. The empirical model for the meta-regressions reported in Table 1 is:

1 $$Y = \beta_{0} + \beta_{1} CBFM\_Duration + \beta_{2} Dry\_Moist + \beta_{ 3}Stated\_{\mathrm{Re}} vealed + \beta_{4} Pub\_Year + \beta_{5} Par\_Com + \beta_{6} Case\_{\mathrm{Re}} gion + \beta_{7} Sample\_Size + \epsilon$$

where *β*_0_ is the intercept, *β*_1_, *β*_2_,…, *β*_*n*_ are the coefficients of the independent variables, and *ϵ* is the error term. The dependent variable *Y* is the t-statistic of a predictor of participation, and X represents the characteristics of the study site and methodology listed in Table 1*.* Summary statistics for the dependent variable (t-statistics) are shown in Table 2.

**Table 1:** Description of study level characteristics and methodological factors as independent variables

Independent variables	Type	Description
***Characteristics of case site***		
Duration of community-based forest management (CBFM_Duration)	Continuous (years)	Calendar year when the CBFM was initiated (established) in the case region or country
Community vs. participatory (Com_Par)	Binary (1 = Community)	Level of devolution in CBFM based on forest management and use rights and responsibility transfer to local people: community forestry with higher devolution of rights to local people and participatory as relatively lower transfer of rights to local people
Case region (Asia_Other)	Binary (1 = Asia, 0 = others)	Classifies case regions into Asia (1) and others (0), where the "others" category includes East, West, and Central Africa, as well as North America
Dry vs. moist climate (Dry_Moist)	Binary (1 = Dry)	Climate condition of studied region
***Characteristics of the study methodology***		
Stated vs. revealed participation (Stated_Revealed)	Binary (1 = Stated)	Stated participation is the willingness to participate, while revealed participation is reported participation in CBFM
Publication year (Pub_Year)	Continuous (calendar year)	Publication year of the study
Sample size (Sample_Size)	Continuous (number)	Total number of participants/respondents in the study sample

**Table 2:** Summary of dependent variables used in meta-regression model

Description	Obs	Mean (St. Dev)	Min–Max
***Dependent variables are t-statistics of coefficients on following independent variables in regressions on participation:***		***T-statistic value***	
Distance to forest	34	− 0.35 (1.87)	− 6.4 to 1.79
Household head age	32	0.39 (1.81)	− 4.05 to 7.5
Household size	43	0.47 (1.86)	− 6.0 to 3.4
Land size	50	0.70 (2.17)	− 5.0 to 6.0
Respondent age	29	0.50 (2.32)	− 3.45 to 6.2
Respondent education	60	0.98 (2.07)	− 3.9 to 6.02
Respondent gender	65	1.15 (1.8)	− 4.7 to 6.2

## Results

### Synthesis of studies on participation in CBFM under meta-analysis

We identified 47 studies with 66 cases of participation in community forestry in North America, South Asia, Southeast Asia, the Middle East, East Africa, Central Africa, and West Africa (Supplementary Material Fig. S1A). We found that most studies had been conducted in South and Southeast Asia, including Nepal (12 studies), India (9 studies), Indonesia (3 studies), and Thailand (2 studies); we also found multiple studies in Ethiopia (6 studies), Kenya (6 studies), Ghana (5 studies), and Burkina Faso (6 studies). We found that 70% of the studies were conducted within the last decade (Supplementary Material Fig. S1B). Almost all (95%) of the studies employed logistic and probit models along with linear probability models (Supplementary Material Table S1). The primary method of data collection was cross-sectional household surveys, which yielded 20,306 responses summed across the 47 studies.

### Independent variables (predictors) investigated in studies of participation in CBFM

We found 248 distinct predictors used to model people's participation in CBFM globally (see Supplementary Material Table S2 for details). These predictors were tested 1034 times across cases. Social predictors were the most frequently investigated (479 times), followed by economic (217 times), biophysical (136 times), and governance (124 times) predictors (Table 3). Based on the proportion of all cases that included a statistically significant governance variable and the proportion of times that an included governance variable was statistically significant, the governance category ranked first in terms of statistical power (Table 3). The proportion of times that an included variable had a statistically significant effect on participation was also high for benefits of participation (56%), economics (51%), institutional arrangements (48%), biophysical (46%), and social (42%). Predictors representing the theory of planned behavior are the least frequently investigated and are not significantly associated with participation.

**Table 3:** Vote count results by category of predictors describing people’s participation in community-based forest management. Sig +  = positively significant; InSig = Insignificant; Sig- = negatively significant; % Sig = percent significant

Category		Number of predictors		Sig +	InSig	Sig-	Total times used	% included	% Sig (cases that included predictors)	% Sig (all cases)
Benefits from participation		10		15	12	0	27	4	56	2.5
Biophysical		28		37	74	25	136	11	46	5
Economic		58		71	106	40	217	23	51	12
Governance		66		67	41	16	124	27	67	18
Institutional arrangement		9		13	18	5	36	4	50	2
Social		72		139	279	61	479	29	42	12
Theory of planned behavior		5		1	14	0	15	2	7	0.15
Totals		248		343	544	147	1034			

The second column in Table 3 displays the total number of distinct predictors per category, while the seventh column (% included) displays the percentage of cases that incorporate these predictors. We found that cases frequently included at least one predictor from the economic, governance, and social categories, accounting for 79% of the cases. The eighth column shows the proportion of predictors that were significant within each category when included in a case, where the governance (67%) has a highest proportion followed by economic (51%), and benefit from participation (56%) categories, while the social category has a notably low rate of statistical significance at 42%. However, since researchers often prioritize significant predictors, potentially overlooking other valuable insights (Barker et al. 2021), the rate of statistical significance of the included predictors does not reveal the full story (Pattanayak et al. 2003). The last column of Table 3 (the product of the numbers in the seventh and eighth columns) shows that the percentage of times a predictor was statistically significant among all cases. The percentages in this column do not suggest the same rank ordering of categories as in the eighth column, suggesting the possibility of publication bias.

Looking at how often predictors were tested and their statistical significance, we found that only a minority of predictors in each category were tested more than 10 times, with most of those achieving statistical significance approximately 40–45% of the time (Supplementary Material Fig. S2). In contrast, the predictors tested fewer than ten times exhibited a wider range of percent significance, with some achieving statistical significance 100% of the time. This pattern suggests that the more often a predictor is tested, the less likely it is to show highly significant rates, supporting the idea that repeated testing across diverse contexts tends to normalize results and reduce bias. For instance, within the biophysical, economic, and social categories, we observe that each includes a commonly tested predictor such as distance to forest (35 times), land size (50 times), and gender (65 times), and each of those is significant around 45% of the times included.

### Consistently explored independent variables (common predictors) to describe people’s participation in CBFM

**Fig. 2:**
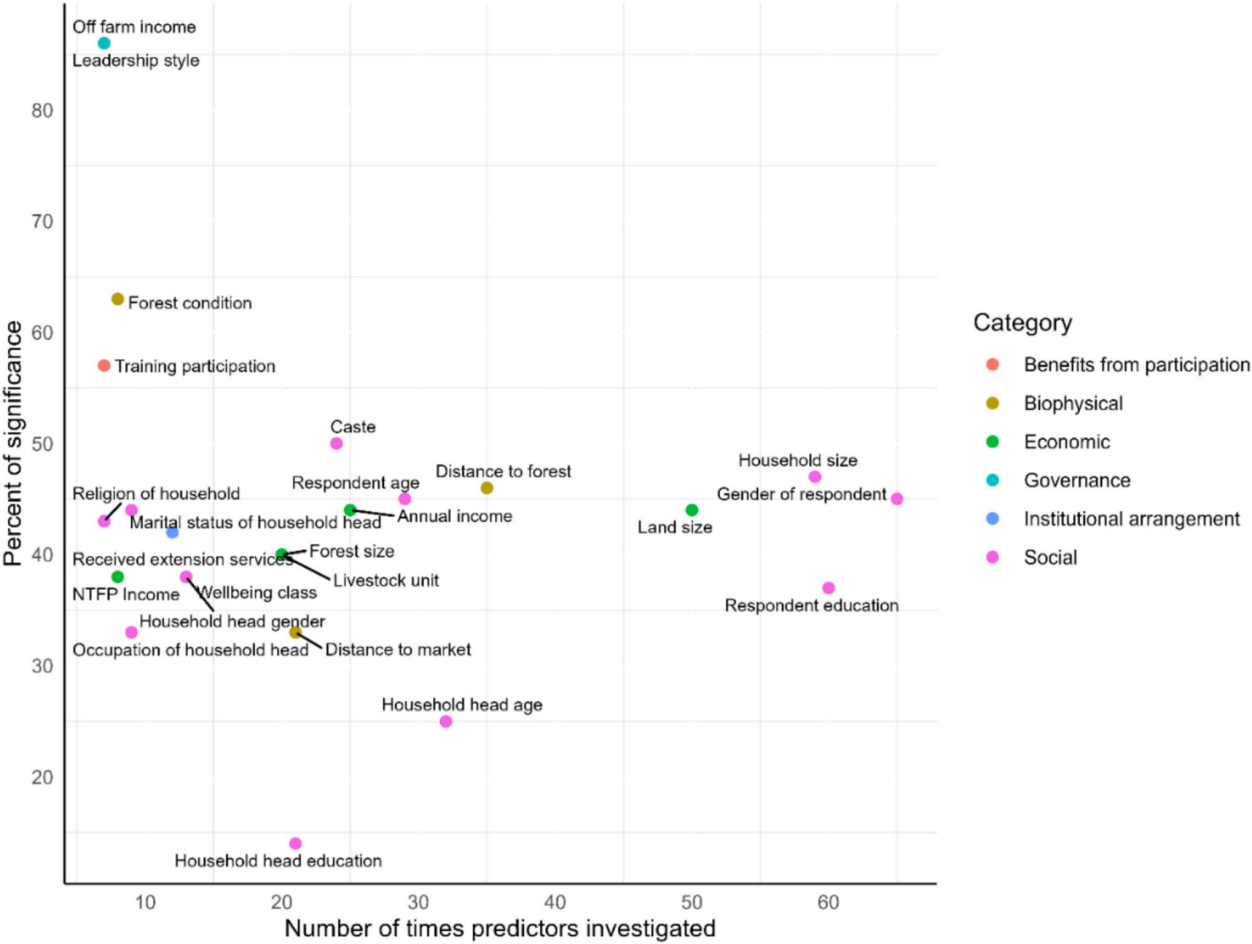
Consistently investigated predictors (≥ 7 times) grouped by category, showing percentage of significant association (either direction) with people’s participation in CBFM. Each point represents a predictor, and each color represents a category. Some predictors are represented by the same dot because they have the same usage frequency and significance. Specifically, off-farm income (economic) and leadership style (governance) overlap; forest size (biophysical) and livestock unit (economic) overlap; and wellbeing class (economic) overlaps with household head gender (social)

We found 24 predictors that were consistently included (≥ 7 cases) in the participation models. More than 60 cases included social predictors such as household size, gender, and education (Fig. 2). Researchers investigated land size, annual income, and livestock units 50, 25, and 20 times, respectively, as economic factors. Biophysical predictors, such as the distance to the forest, were tested 35 times, whereas the distance to the market was tested 21 times. While researchers clearly consider these variables important to include in their model, they have statistically significant effects in only approximately 45% of the models in which they are included. Although included less frequently, predictors such as training participation, forest condition, off-farm income, and leadership style are more frequently statistically significant, indicating stronger associations with people's participation.

### Independent variables with highest significant association with participation

We found that most statistically significant predictors had a consistent positive or negative effect. For instance, off-farm income is consistently associated with a lower probability of participation. Better forest conditions, receiving training from the CBFM, cooperation from forest authority to users, the level of forest resource support to users from the CBFM, CBFM leadership quality (high or low) perceived by households, and the level of social cohesiveness are all consistently associated with a higher probability of participation (Table 4). In contrast, we found that the type of leadership style (manipulative, authoritarian, participative, and charismatic) and private land tenure were consistently associated with the probability of participation when included in the model but with different patterns of association in different cases.

**Table 4:** Key predictors that have the highest proportion of statistically significant associations with local participation in CBFM (≥ 50%). % Sig (included cases) = describes the percentage of times the predictor is significant when included in the studies and % sig all cases describe the percentage of times the predictor is significant in all study cases (out of 66 cases)

Predictors	No. of times modeled	% Sig (included cases)	% Sig (all cases)	Sign ( ±)
***Benefits from Participation***				
Participated in training from CBFM	7	57	6	+
***Biophysical***				
Better forest condition	8	63	7.6	+
***Economic***				
Household annual expenditure	6	67	6	+
Land tenure type (Private land)	5	80	6	±
Having off farm income	7	71	7.6	−
Fraction of income derived from forests	6	100	9	±
***Governance***				
Type of leadership style	7	86	9	±
Co-operation from forest authority to users	4	100	6	+
CBFM Leadership quality perceived by households	4	100	6	+
***Institutional arrangement***				
Involvement in Executive committee	6	50	4.5	±
***Social***				
Perception on community forest species distribution	6	50	4.5	+
Caste of Households head	24	50	18	±
Resource support to users from CBFM	4	100	4.5	+
Level of social cohesiveness	4	100	4.5	+
Years of residency	6	67	4.5	±

### Independent variables category describing stated and revealed participation

**Fig. 3:**
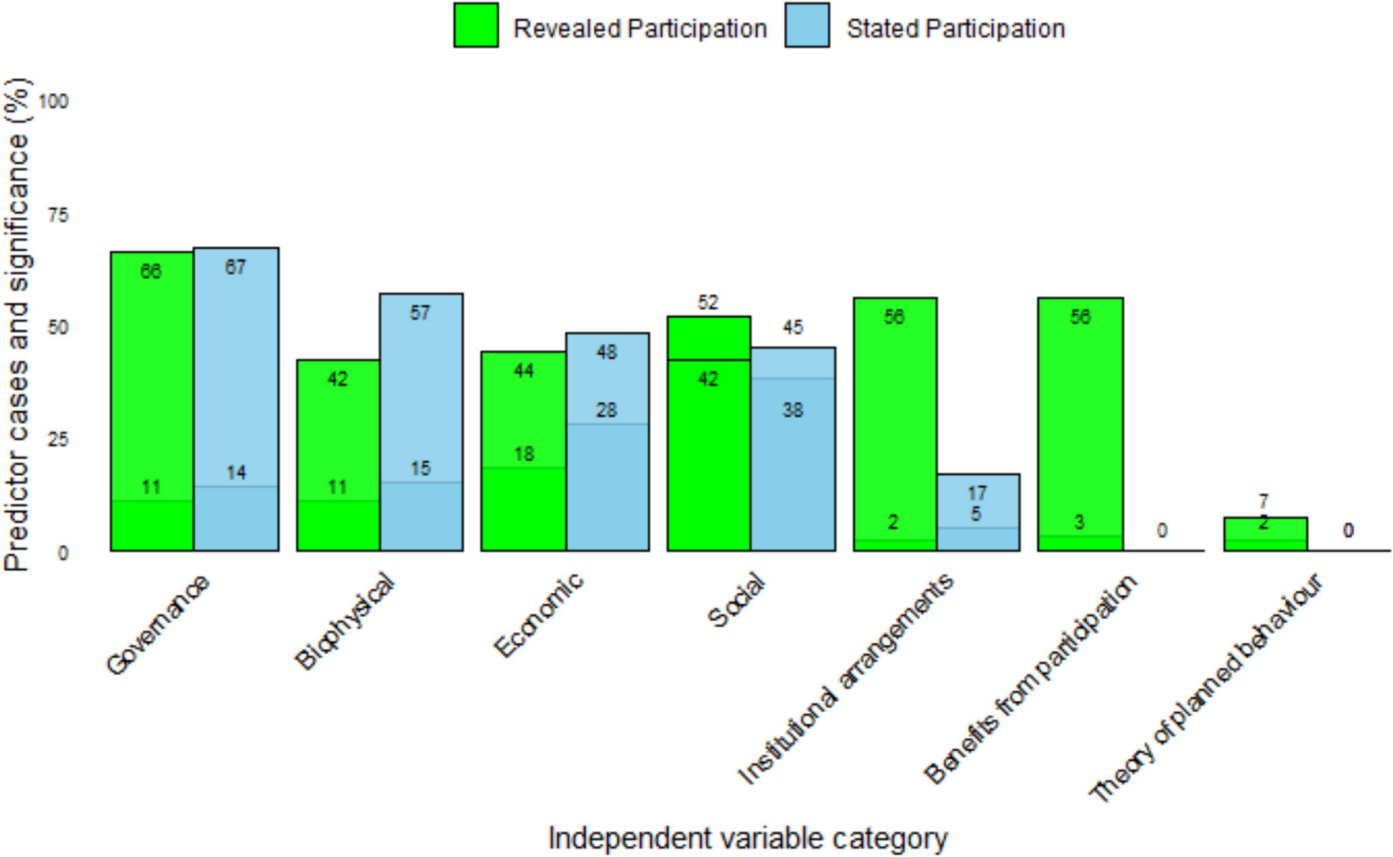
Independent variable categories for both stated and revealed participation. The lower number in each category bar represents the percentage of times predictors from each category were used to model participation out of total number of times predictors used across all categories. On the other hand, the upper number in the bar signifies a statistically significant association. An exception is the revealed participation in the social category, where the positions are reversed: the upper number (52%) reflects the percentage of times predictors were used, and the lower number (38%) shows the percentage of significant associations

We compared stated and revealed participation using two key metrics for each category: (1) the percentage of times predictors from each category were used (relative to the total number of times predictors were used across all categories), and (2) the percentage of times predictors within each category showed statistically significantly associated with participation in CBFM. For instance, in the analysis of revealed participation in the governance category, we found that 11% of the predictors of participation were from the governance category, of which 66% showed a statistically significant association (Fig. 3).

Across models of both stated and revealed participation, economic and social predictors were the most commonly included. In about 45% of the cases in which they were included, economic and social predictors were significantly associated with participation. Governance, social, and economic predictors do not exhibit any significant differences between the two types of participation, regardless of the predictors used, and they have the strongest significant association with participation (Fig. 3). Biophysical predictors are more likely to be significantly associated with stated participation than the revealed participation, even though the number of cases appears to be comparable. Predictors in categories such as institutional arrangements and participation benefits are more likely to exhibit a statistically significant relationship with revealed measures of participation (56% of revealed participation cases) than with stated measures of participation.

### Meta-analysis of prevalence to estimate predictor strength and analyze publication bias across all studies and consistently tested independent variables

**Fig. 4:**
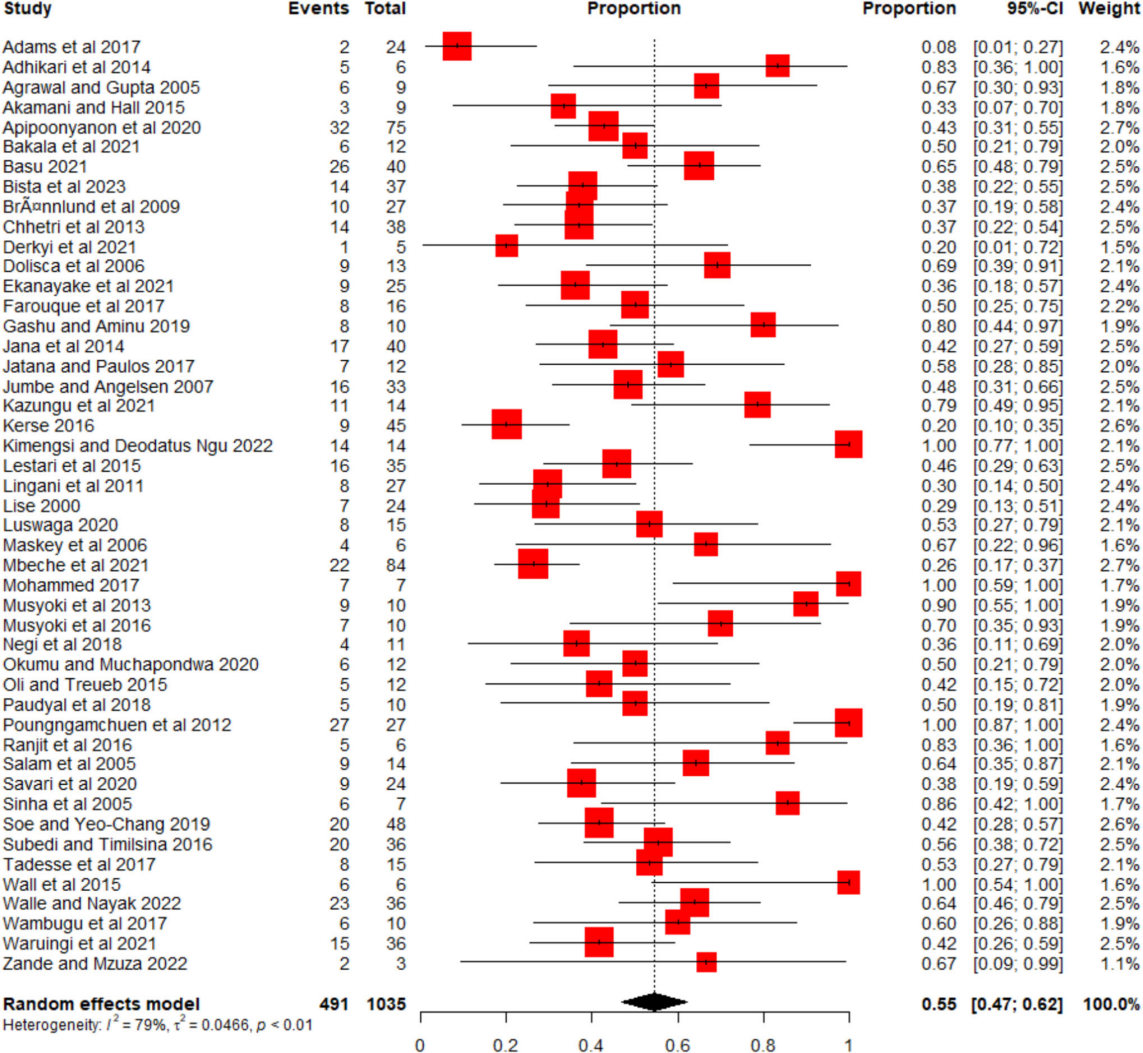
Forest plot, summarizing the meta-analysis of 47 studies, showing the proportion of significant predictors of people's participation in CBFM. The “Event” and “Total” columns represent the number of significant predictors and the total number of predictors tested in each study, respectively. Each red square represents the proportion of significant predictors in a study, with its size reflecting the study’s weight in the analysis (based on inverse-variance weighting). Horizontal lines show the 95% confidence intervals, with longer lines indicating greater uncertainty. The diamond at the bottom represents the pooled estimate across all studies, with its width denoting the confidence interval.

The results are summarized in the forest plot (Fig. 4), which shows the prevalence proportion of significant predictors in each study alongside the pooled estimates. On average, 55% of the predictors across all studies were significantly associated with participation in CBFM (95% CI: 0.48–0.62). Studies with a higher proportion than 55% are above average. We found substantial heterogeneity among studies (I^2^ = 79%), likely driven by differences in study context, methods, or time (Barker et al. 2021). Evidence of publication bias was found based on the asymmetry in the funnel plot (Supplementary Material Fig. S3) and confirmed by Egger’s test (t = 3.47, p = 0.001).

**Fig. 5:**
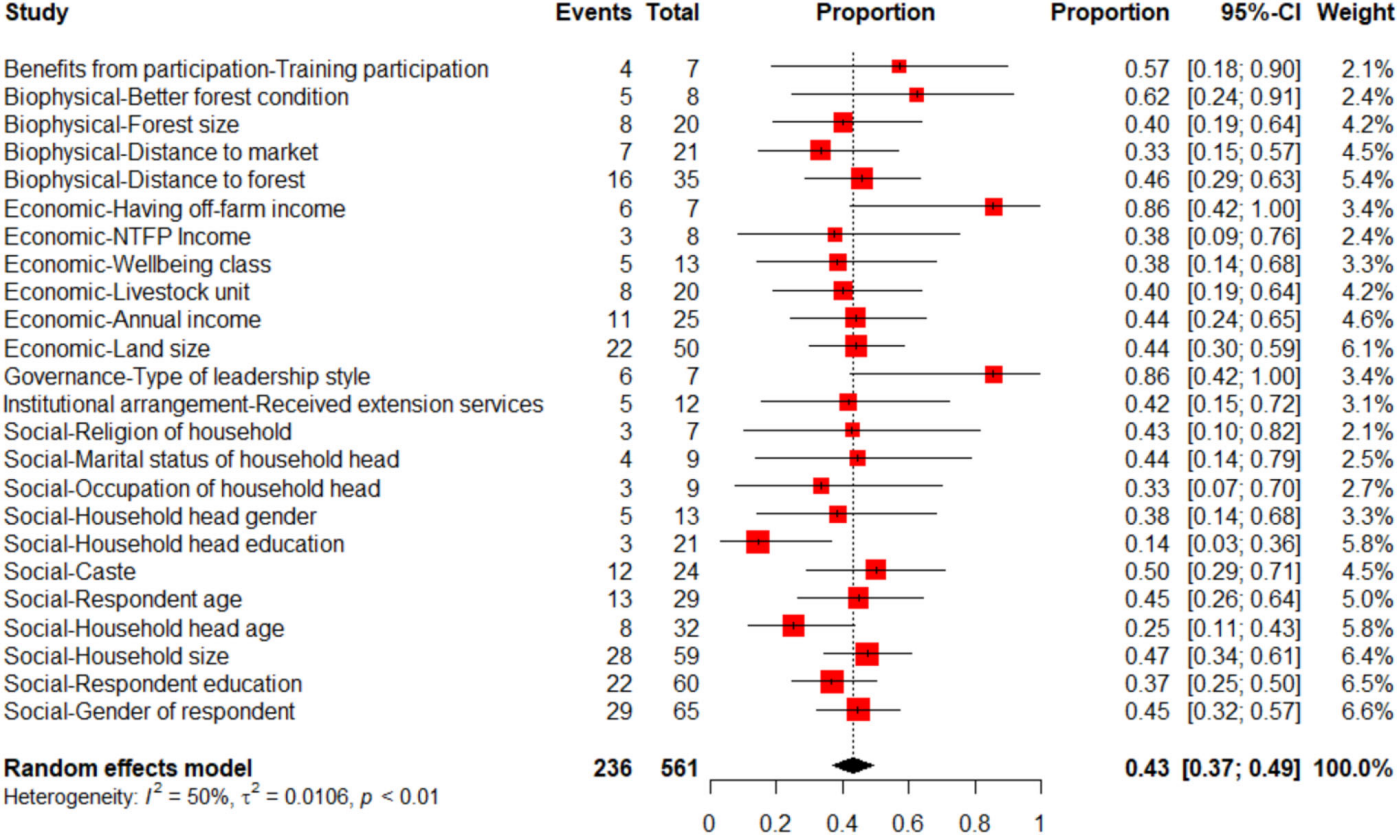
Forest plot showing the proportion of 24 consistently tested predictors of people's participation across the total times each variable was tested. The “Predictors” column lists each predictor, while the “Events” and “Total” columns indicate the number of times the predictor was statistically significant and the total number of times it was tested, respectively. Each red square represents the proportion of significant results (effect size) for a given predictor with horizontal lines showing 95% confidence intervals. The diamond at the bottom represents the overall or pooled effect size estimate across all predictors with its width representing the confidence interval. The size of the square reflects the predictor’s weight in the meta-analysis, calculated using inverse-variance methods

Similarly, among the 24 consistently tested predictors,^4^ the average effect was 0.43, indicating that, of the 24 consistently tested predictors, there was a statistically significant association with participation in CBFM 43% of the time (Fig. 5). The heterogeneity was 50%, indicating moderate variability between the independent variables. Closer analysis of each predictor reveals variation in their statistical significance; for instance, predictors such as household head education (14% significant) and age (25% significant) are often evaluated but seldom statistically significant. In other words, these predictors had a lower probability of describing participation. Off-farm income (71% significant), type of leadership style (86% significant), and forest condition (63% significant) are seldom included but are likely to be statistically significant (Fig. 5). Assuming no publication bias, these predictors are relatively more important and likely to lead to more or less participation in CBFM. The almost symmetrical distribution of the funnel plot (Supplementary Material Fig. S4) and the Egger test results indicated no publication bias for the consistently tested predictors (t = 1.5, p = 0.15).

### Meta-regression of consistently tested predictors

We found that the effects of some predictors vary with institutional context, specifically years since the CBFM was established and type of forest management institution (Table 5). These consistently affect the statistical significance (t-statistics) of the coefficients estimated for distance to forest, household head age, land size, and respondent age. As expected, sample size is also positively related to statistical significance. The lack of explanatory power of other methodological features increases confidence in credibility to the results. Other predictors are statistically significant for only one predictor and one type of dependent variable.

**Table 5:** Meta-regression model of frequently used predictors. Predictors that were used more than 30 times are included in this analysis. Statistical significance: *0.1, **0.05, ***0.01. AME: Average marginal effect; Standard errors are presented in parentheses (Here Com_Par = Community vs Participatory; Pub_Year = Publication Year)

Variables	Distance to forest		Household head age		Household size		Land size		Respondent age		Respondent education		Respondent gender	
	AME	x̄	AME	x̄	AME	x̄	AME	x̄	AME	x̄	AME	x̄	AME	x̄
***Characteristics of case site***														
*Intercept*	*450.2 (176.3)*		− *0.04 (0.02)*		− *0.02 (0.01)*		*0.022 (0.01)*		*485 (450.2)*		− *0.02 (0.94)*		− *0.84 (0.94)*	
CBFM_ Duration	− 0.12* (0.06)	1997	0.34*** (0.10)	1996	0.06 (0.07)	1992	− 0.12* (0.06)	1995	− 0.02 (0.06)	1996	− 0.02 (0.04)	1995	− 0.01 (0.04)	1994
Com_Par	0.83 (1.63)	0.12	8.18** (3.36)	0.09	− 0.15 (1.14)	0.23	− 2.8** (1.40)	0.16	− 2.13 (1.42)	0.21	− 1.3 (1.24)	0.08	− 0.46 (0.99)	0.18
Case_Region	− 0.07 (1.06)	0.32	1.44 (1.43)	0.40	1.6 (1.44)	0.40	− 0.07 (0.88)	0.52	2.97** (1.36)	0.52	0.37 (0.65)	0.53	− 0.24 (0.61)	0.52
*Dry_Moist*	1.72* (0.97)	0.45	1.08 (1.25)	0.40	0.83 (1.00)	0.26	0.48 (0.78)	0.38	1.49 (1.11)	0.26	0.88 (0.62)	0.43	0.96 (0.61)	0.32
***Characteristics of the study methodology***														
Stated_ Revealed	3.06 (2.26)	0.06	1.57 (0.06)	0.22	2.28 (1.65)	0.07	2.63 (1.69)	0.06	− 1.52 (2.76)	0.07	− 1.15 (0.93)	0.13	1.58* (0.85)	0.12
Pub_Year	− 0.08 (0.08)	2018	− 0.16 (0.12)	2016	0.05 (0.06)	2014	0.01 (0.08)	2017	− 0.22 (0.20)	2018	0.10** (0.04)	2015	0.06 (0.04)	2015
Sample_Size	0.004** (0.00)	358	0.00 (0.00)	389	0.00 (0.00)	263.4	0.00 (0.00)	362	0.00* (0.00)	291	0.00 (0.00)	311	0.00 (0.00)	294

If we look at the relationship between the effect of land size and forest management institutions, the results show that larger landholdings are more likely to be associated with lower participation in community forestry institutions than in participatory institutions. Similarly, households with a higher household head age are positively associated with higher participation in community forestry institutions but not in participatory institutions. These results indicate that the type of forest management institutions (participatory vs. community) significantly influences the relationship between predictors and participation in CBFM. Similarly, a strong positive association between the effect of the age of the household head and CBFM duration implies that, as the CBFM program ages, the household head’s age becomes a more important determinant of participation. The effect of the age of the respondent was positively correlated with the case region. The respondent's age plays an important role in CBFM participation in the regions where there is a relatively longer and more successful history of CBFM than in other regions.

We found that in stated preference studies, which may capture the individual respondent's preferences more than the household's behavior patterns, the gender of the respondent matters the most. More recently published studies are more likely to find that education has a positive and significant effect on participation.

## Discussion

### Governance as a key category in a rich predictor’s landscapes

Our meta-analysis of 47 studies worldwide identified 248 predictors of people’s participation in CBFM, demonstrating the richness and complexity of the factors influencing local engagement. We grouped the predictors into seven categories, with the strongest evidence linking participation to social, governance, economic, and biophysical categories.

Among these, governance emerged as the most significant, consistently associated with participation in 67% of instances with over 80% of governance predictors showing a positive effect. This emphasizes the critical role of effective governance structures in fostering people’s participation in CBFM (Agrawal 2001; Poudyal et al. 2023). Effective CBFM governance, through inclusive decision-making, benefit sharing, training, leadership, technical support, institutional regimes, and forest management plans, ensures local people’s participation in planning, implementation, and monitoring of sustainable forest management, thereby advancing social equity and community empowerment (Pokharel and Tiwari 2013; Lamichhane and Parajuli 2014; Kimengsi and Bhusal 2021). Comparative evidence also supports governance quality as a key determinant of participation (Maraseni et al. 2019). In our study, the governance category consisted of 65 predictors related to the processes and implementations of CBFM. These predictors include decision-making, benefit sharing, attending meetings, capacity-building training, leadership styles, technical assistance, the roles of forest authorities, types of institutional regimes, and the effectiveness of forest management plans. Our findings show governance predictors remained significant even after accounting for publication bias, confirming their fundamental role, especially as global forest governance becomes more complex and multiscalar (Arts et al. 2024).

Similarly, the category of economic predictors, particularly direct benefits and incentives, is also found to have strong positive relationships with local participation in forest management (Chhetri et al. 2013; Zande and Mzuza 2022). Social predictors, such as household size, gender, respondent education level, and age, were among the most frequently included but least often statistically significant. Their frequent inclusion in the models may be due to their easy availability from household surveys and census data.

Further, biophysical predictors such as better forest condition, forest size, distance to the forest, and market access also shape people's participation (Epstein et al. 2024). For instance, people are more likely to participate when forest conditions are better. These significant predictors across various categories reinforce that CBFM operates within interlinked social, economic, and ecological systems (Ojha and Hall 2021; Hajjar et al. 2021). Geographically, studies included in our analysis were concentrated in South Asia and East Africa, particularly Nepal, India, Kenya, and Ethiopia, where CBFM has expanded rapidly in the last few decades (Gilmour 2016). While we did not compare predictors across regions, results underscore regional heterogeneity in research efforts and the growing recognition of community participation in forest management, as reflected in the surge of studies over the past decade (Smith et al. 2023).

### What explains people’s participation in CBFM?

We identified 15 predictors that showed a significant association with people's participation in CBFM in more than 50% of the cases (each modeled more than five times), and 5 predictors that showed a significant association in all cases (100%) when modeled at least four times (Table 4). These findings offer important insights for designing more responsive and inclusive participatory forest management strategies. For example, off-farm income of households is consistently associated with a lower probability of participation. Rising off-farm income stabilizes household finances and reduces forest reliance (Eknayake et al. 2021), while encouraging market-oriented livelihoods that divert attention from forest activities (Gentle and Thwaites 2016; Laudari et al. 2024). In Nepal’s mid-hills, remittance-driven off-farm income has reduced participation in community forestry (Bista et al. 2023). CBFM has also been criticized for providing mainly subsistence benefits, limiting its economic relevance (Bhusal et al. 2020). These patterns suggest that CBFM must adapt to shifting household priorities by integrating livelihood-relevant incentives.

Additional predictors such as improved forest conditions and higher total annual household expenditure consistently demonstrated positive associations with participation. Likewise, predictors such as the cooperation of forest authorities, the perceived leadership quality, institutional support for forest resources, and social cohesiveness demonstrated a 100% positive association with participation. Studies emphasized the positive impact of access to training, technical assistance, and forest extension services on enhancing people's participation (Tadesse et al. 2017; Bakala et al. 2021; Bista et al. 2023). Additionally, supportive forest institutions, institutional rules, and arrangements are also critical for and collective action (Agrawal et al. 2023), whereas the absence or limited provision of training and technical assistance hinders participation (Dolisca et al. 2006; Apipponyanin et al. 2020). These findings reinforce that strengthening institutions, providing training, and ensuring transparent governance are essential for sustained CBFM engagement across spatial and ecological contexts.

### Factors to be considered when designing a CBFM

CBFM policies must adapt to changing socioeconomic and ecological conditions (Ojha et al. 2016; Paudyal et al. 2023; Laudari et al. 2024) and address the needs of underprivileged and marginalized groups who rely on forests for their livelihood (Subedi and Timilsina 2016. Protective forest measures often reduce harvest-based benefits, diminishing economic incentives for collective action (Dokken and Angelsen 2015).

Institutional factors such as leadership style and land tenure were also consistently influential. Positive, participative and charismatic leadership foster participation, while manipulative or authoritarian leadership discourages it (Sinha and Suar 2005; Poungngamchuen and Namvises 2012). Effective leadership enhances ownership, trust, and reciprocity (Kimengsi et al. 2019). Land tenure also mattered: inherited land tenure was positively associated with participation in 80% of cases, consistent with evidence linking secure tenure to stronger CBFM engagement (Ratsimbazafy et al. 2012; Wambugu et al. 2017). Thus, participation in CBFM can be promoted through both inclusive leadership development and secure equitable land tenure arrangements.

Among household characteristics, caste was consistently significant in countries where it is relevant. Some studies found that households from lower castes (Dalit, Janajati, or marginal) are more likely to participate due to their dependency on forests (Bista et al. 2023), while others found lower participation of marginalized groups due to competing livelihood demands (Maskey et al. 2006; Subedi and Timilsina 2016). This contradiction underscores the need for participatory approaches that consider the competing livelihood demands of marginalized groups.

We also identified 24 predictors consistently used (investigated ≥ 7 times) to model people's participation in CBFM, with 13 of them examined over 20 times (Fig. 2). On average, they were statistically significant 43% of the time. However, the meta-analysis of consistently tested predictors revealed differences in their use and their relationship with participation. For instance, demographic predictors such as education and age were rarely significant, despite their frequent inclusion in quantitative models. Researchers should reconsider reliance on these variables and prioritize less frequently tested but consistently significant predictors such as off-farm income, leadership style, forest condition, and training, which better capture the dynamics of participation.

### Meta-regression

The meta-regression results show that a one-size-fits-all approach to promoting participation in CBFM is not appropriate. Institutional and contextual factors such as the duration of CBFM establishment, type of forest management institution, and region shape which predictors are statistically significant (Epstein et al. 2024). For instance, the age of the household head is more likely to be significant in community forestry, while land size is more influential in participatory forest management systems. These findings highlight that participation, and thus the success of CBFM institutions, depends on tailoring policies and strategies to specific regional, institutional, and local contexts.

Methodological factors could also influence results. Significance varies with year of publication, how participation is measured (stated vs. revealed), and sample size. Not all variation reflects policy-relevant heterogeneity. However, temporal shifts may capture the evolving nature of CBFM in response to changing socio-economic and ecological conditions (Ojha and Hall 2021; Laudari et al. 2024; Agrawal et al. 2023). This suggests that the drivers of people’s participation themselves can change over time. Policymakers should recognize these dynamics, while researchers should prioritize longitudinal analyses to track how predictors of participation shift in different contexts.

### Publication bias

We found evidence of publication bias at the study level, which indicates the variation in predictor effects may influence publication decisions. Positive and statistically significant predictors are more likely to be published (Thornton and Lee 2000; Atmadja and Sills 2015). For instance, studies such as Tadesse et al. (2017), Poungngamchuen and Namvises (2012), Mohammed (2017), and Kimengsi and Deodatus Ngu (2022) demonstrate this finding that all the predictors are significant with an effect size of one. However, the publication bias test for consistently tested predictors revealed no bias, suggesting that these variables are routinely used as standard components for modeling participation in CBFM. Further, lack of publication bias indicates that these predictors are genuinely important and warrant continued study.

### Limitations and extended use of vote-count meta-analysis

Due to its key focus on statistical significance, vote count meta-analysis often lacks insight into the magnitude or direction of relationship between independent and dependent variables (Floress et al. 2019). Nonetheless, prior studies revealed that results of vote count methods often align with meta-analyses using effect sizes (Prokopy et al. 2008; Baumgart-Getz et al. 2012). We therefore used vote count analysis as a starting point to identify significant associations among predictors based on the count of significant associations. Beyond this traditional use, we also analyzed the strength and direction of the key predictors by examining their frequencies of associations. We used these results in a meta-analysis of prevalence to determine the weighted average proportion estimate of factors associated participation at both the study and predictor levels, alongside for publication bias.

We further utilized meta-regression to examine how study-level characteristics and methodological factors influence observed effect sizes (Atmadja and Sills 2015). By analyzing variations in t-statistics across studies, we identified reasons for significant differences in the results and their impact on relationships between frequently used predictors and people's participation in CBFM. This combined approach provides a more comprehensive understanding of the drivers of participation by addressing both the magnitude and direction of effects while accounting for potential biases in the literature.

## Conclusions

Drawing on 66 cases from 47 studies across 18 countries, this study synthesizes global quantitative evidence to identify key predictors of people’s participation in community-based forest management (CBFM). We categorized 248 predictors into seven groups and assessed their generalizability using vote-count meta-analysis, meta-regression, and publication bias tests.

Fifteen predictors showed statistically significance association in more than 50% of cases, with five showing 100% significant association, either positively or negatively. Negative predictors included off-farm income while positive predictors included cooperation of forest authorities, perceived quality of CBFM leadership, institutional support for forest resources, and social cohesiveness. Leadership style, land tenure status and fraction of household income from forests exhibited mixed effects, emphasizing the importance of effective leadership and secure land rights. Notably, many predictors that are commonly used rarely show statistical significance. Their common use might be more linked to their easy accessibility in household surveys or census data than to their importance to CBFM participation.

Future research efforts should explore alternative factors, such as off-farm income, leadership style, forest condition, and training participation, which have shown a higher likelihood of being statistically significant in explaining participation in CBFM. The absence of publication bias among the frequently used predictors indicates that these predictors are genuinely important factors that deserve further study. Overall, these results highlight the multifaceted and context-specific nature of participation in CBFM. Policymakers and practitioners should design interventions tailored to local conditions while emphasizing predictors most reliably linked to participation. Strengthening CBFM outcomes requires integrating household- and community-level strategies that foster inclusive, adaptive, and effective forest governance.

## Supplementary Information

Below is the link to the electronic supplementary material.

Supplementary file1 (PDF 924 KB)

## Footnotes

1 Search used: #1: Factors affecting people participation in community forestry or collaborative forest management or joint forest management or village forestry, sustainable forest management or CBFM, or forest conservation; #2: Determinants of people’s participation in community forestry or collaborative forest management or joint forest management or village forestry, sustainable forest management or CBFM or forest conservation; #3: People participation in community forestry or collaborative forest management or joint forest management or village forestry, sustainable forest management or CBFM or forest conservation; #4: Willingness to participate in CBFM and #3; #5: level of participation and #3; #6: Participation behavior and #3. #7: Incentives for CBFM.

2 The meta-analysis encompasses 66 cases from 47 studies. "Study" refers to the individual articles under meta-analysis, while "case" refers to the different empirical models used in each of these studies to analyze variables that determine community participation in CBFM. Some studies contributed more than one case, with two or more empirical models analyzed from a single publication. For instance, one study may have different models that analyze the socioeconomic, institutional, and governance factors influencing CBFM participation, and each is considered a case even if it came from the same study. Predictors tested in more than seven studies were classified as consistently included variables. The threshold of seven was selected to balance inclusivity with analytical robustness, ensuring that a sufficient number of predictors were retained for comparison without over representing rarely studied variables.

3 In this study, the effect size quantifies the strength and consistency of predictors for each study case at the study level. We achieve this by assessing the proportion of significant predictors of people's participation (total significant events) across all tested independent variables. Similarly, we measure the proportion of total significant events across the total number of tests for each consistently tested predictor.

4 Off-farm income does not include forest-based income. Forest condition refers to the biophysical state of a community forest, whether it is good or bad. The occupation of the household head refers to the primary occupation of the HHs head, while the caste of the household represents either a higher or lower caste group. Please refer to Supplementary Material Table S1 for additional details about each predictor.
