# Supplementary material for: What drives participation in community-based forest management? Insights from a global review
Source: Ambio. 2025 Nov 27;55(6):1181–97. doi: 10.1007/s13280-025-02278-7 (PMC13125443; doi:10.1007/s13280-025-02278-7)
Supplement: Supplementary file 1 — Supplementary file1 (PDF 924 KB) [file 13280_2025_2278_MOESM1_ESM.pdf]

Supplementary Material

What Drives Participation in Community-Based Forest Management? Insights from Global Review

Prabin Bhusal

PhD student

Department of Forestry and Environmental Resources

North Carolina State University

Raleigh NC 27695 USA

Email: pbhusal@ncsu.edu

And

Assistant Professor

Institute of Forestry,

Tribhuvan University, Pokhara Campus, Hariyokharka-15, Nepal

pbhusal@iofpc.edu

Rajan Parajuli, PhD

Associate Professor (Corresponding author)

Department of Forestry and Environmental Resources

North Carolina State University

Raleigh NC 27695 USA

Email: rparaju@ncsu.edu

Phone: +1-919-515-1553

And

Erin Sills, PhD

Professor and Head

Department of Forestry and Environmental Resources

North Carolina State University

Raleigh NC 27695 USA

Email: sills@ncsu.edu

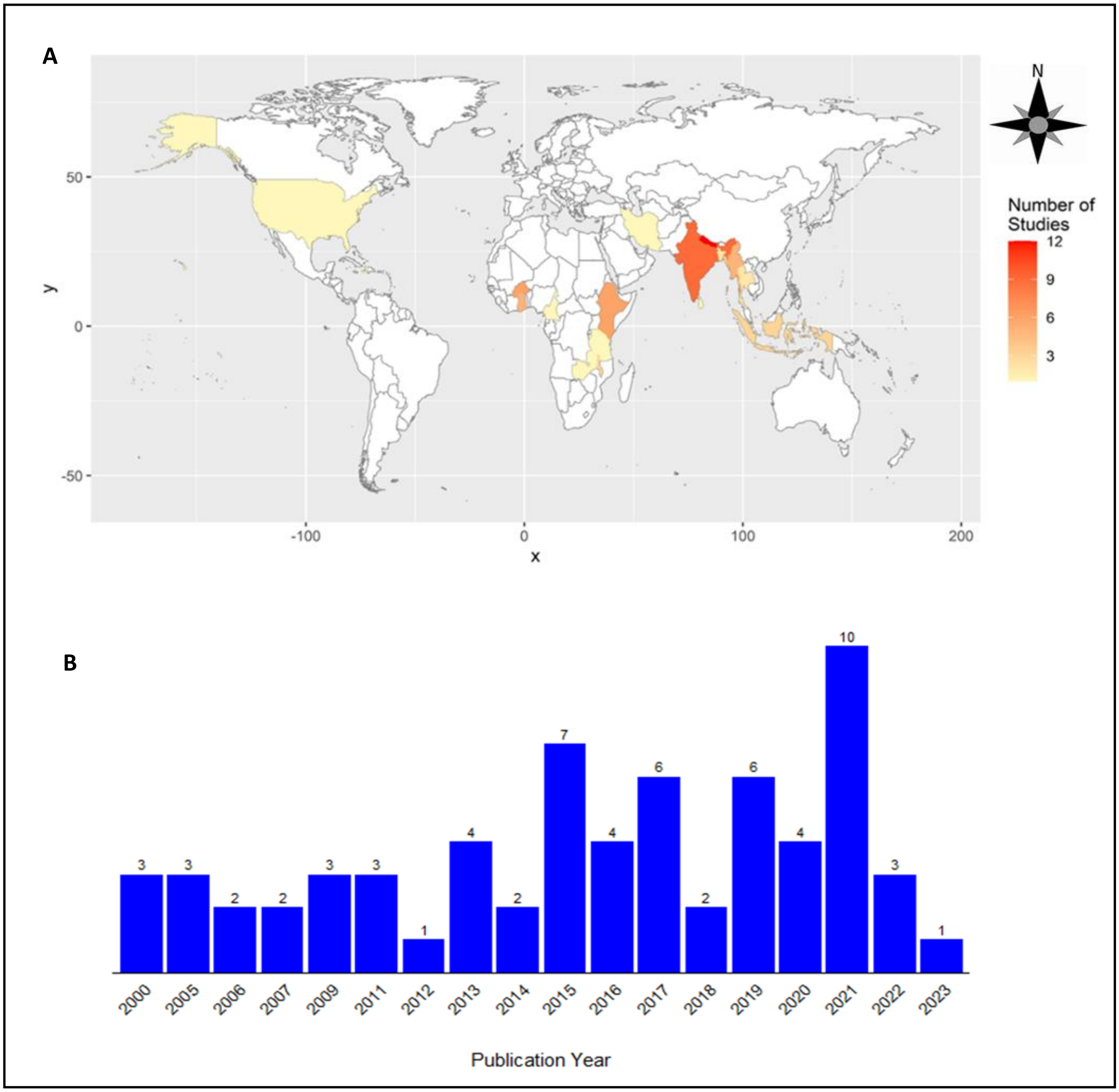

Fig. S1 A: World heat map showing distribution of studies of participation in CBFM, **B:** Publication year of studies of participation in CBFM

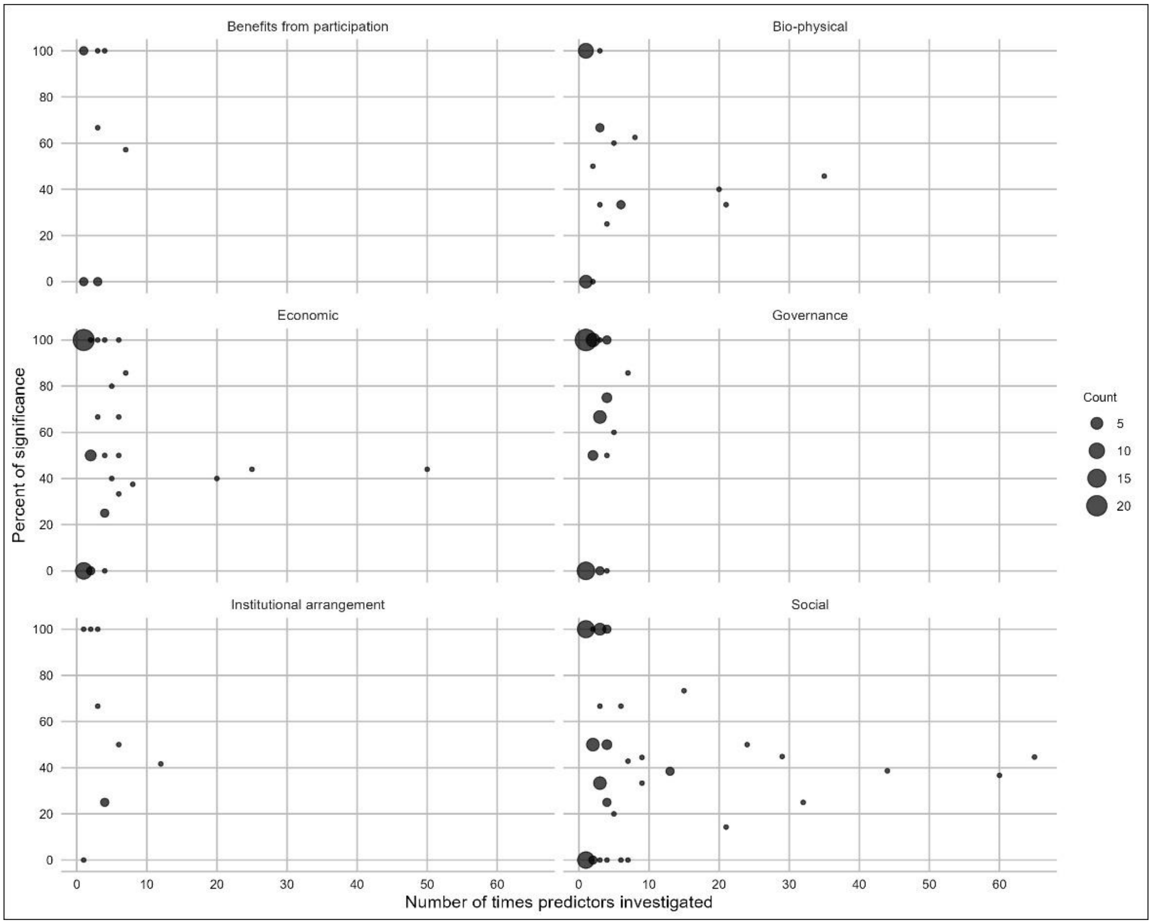

Fig. S2: Scatterplot of the number of times a predictor is investigated and the percent of times it is found to be statistically significantly (either negative or positive) related to local participation in CBFM. Each point represents a unique predictor and larger dots indicate that multiple predictors were tested the same number of times and yielded the same percentage of significance.

Table S1: Characteristics of studies included in the review

Authors and year	Case country	Dependent variable	Analysis model used	Sample size
Adams et al. (2017)	Ghana	Level of participation	Chi square	407
Adhikari et al. (2014)	Nepal	level of participation in common property resource governance	Ordered probit	200
Agrawal & Gupta (2005)	Nepal	Level of participation in community forestry program	Ordered probit	1666
Akamani & Hall 2015	Ghana	Willingness to participate in CFM program	Multiple linear regression	209
Apipoonyanon et al. (2019)	Thailand	Participation in CFM	Logistic regression	400
Bakala et al. (2021)	Ethiopia	Participation in PFM program	Logistic regression	408
Basu (2021)	India	Overall participation index	Multiple linear regression	252
Bista et al. (2023)	Nepal	Participation in management and decision making	Ordered logistic	415
Brannlund et al. (2009)	Burkina Faso	Level of participation in forest surveillance and community work for forest conservation	Multinomial model (discrete choice model)	162
Chhetri et al. (2013)	Nepal	Level of participation of households in decision making activities (meetings, general assemblies)	Ordered logistic	176
Coulibaly-Lingani et al. (2011)	Burkina Faso	Level of participation in decision making	Multiple linear regression	165
Derkyi et al. (2021)	Ghana	Level of participation in SFM monitoring and enforcement	Logistic regression	1200
Dolisca et al. (2006)	Haiti	Level of participation	Multiple linear regression	243
Ekanayake et al. (2021)	Sri Lanka	Participation intention in CF program	Ordered probit and logit models	100
Farouque et al (2017)	Bangladesh	Attitudes towards CBFM approach	Stepwise linear multiple regression	80
Gashu & Aminu (2019)	Ethiopia	Perception on participatory forestry management participation in livelihood support	Logistic regression	128
Jana et al. (2014)	India	Level of participation in JFM	Multiple linear regression	150
Jatana & Paulos (2017)	Ethiopia	Willingness to participate in participatory forest management and its performance	Logistic regression	183
Jumbe & Angelsen (2007)	Malawi	Level of participation	Ordered probit	205
Kazungu et al. (2021)	Zambia	Participation in FS programs	Logistic regression	1123
Kerse (2016)	Ethiopia	Level of participation in forest for carbon sequestration	Logistic regression	146
Kimengsi & Deodatus Ngu (2022)	Cameroon	Sustainable forest program participation	Logistic regression and ANOVA	100
Lestari et al. (2015)	Indonesia	Level of participation in community forestry	Logistic regression	571
Lise (2000)	India	Level of participation in forest management	Multiple linear regression	123

Luswaga & Nuppenau 2020	Tanzania	Participation intensity index	Multinomial logit	159
Maskey et al. (2006)	Nepal	Level of participation in forest management	Ordered probit	45
Mbeche et al. (2021)	Kenya	Participation of local communities forest management programs	Fractional regression	924
Mohammed et al. (2017)	Ghana	Level of participation in SFM	Logistic regression	370
Musyoki et al. (2013)	Kenya	Willingness to participate in CFAs	Chi square	160
Musyoki et al (2016)	Kenya	Level of participation of CFA members	Chi square	160
Negi et al. (2018)	Nepal	Involvement of forest-user households in CFM activities	Generalized least squares and Multiple regression	180
Okumu & Muchapondwa (2020)	Kenya	Level of participation	Logistic regression	518
Oli & Treueb (2015)	Nepal	Level of participation in forest activities	Ordered probit	304
Paudyal et al. (2018)	Nepal	Participation of forest conservation activities	Ordered logistic	162
Poungngamchuen et al. (2012)	Thailand	Extent of participation in CFM project	Multiple linear regression	347
Ranjit (2016)	Nepal	People's participation in forest management and protection	Multiple linear regression	125
Salam et al. (2005)	Bangladesh	Sustained participation of respondents in participatory forestry	Logistic regression	581
Savari et al. (2020)	Iran	Participation in SFM	Hierarchical multiple linear regression	173
Sinha & Suar (2005)	India	Level of participation in community forestry programs	Multiple regression	397
Soe & Yeo-Chang (2019)	Myanmar	Willingness to participate in forest conservation by LMA	Logistic regression	330
Subedi & Timilsina (2016)	Nepal	Participation in formulation and implementation processes with CF	Binary logistic regression	116
Tadesse et al. (2017)	Ethiopia	Participation in PFM program	Logistic regression	172
Wall et al. (2006)	USA	Participation in community forestry program	Multiple linear regression	42
Walle & Nayak (2022)	Ethiopia	Participation in PFM program	Ordered logistic	156
Wambugu et al. (2017)	Kenya	Participation in community forestry program	Logistic regression	202
Waruingi et al. (2021)	Kenya	Level of participation in participatory forest management and PELIS	Poisson regression	880
Zande & Mzuza (2022)	Malawi	Community participation in FM in village forest	Logistic regression	100

Table S2: Description of independent variables (predictors) under different categories used to model participation

Independent variables (Predictors)	Description	# of times modeled	# of times significant	Variables measurement level*
Theory of Planned Behavior				
Subjective norm	Whether or not important people in his/her life approved to engage to CBFM	3	0	Household
Attitude	Users' positive or negative attitude towards participation	3	0	Household
Behavior participation	Respondent observable response to participation in CBFM	3	0	Household
Intention of participation	Whether or not respondents specified the purpose to participation in CBFM	3	0	Household
Perceived behavior control	Perceived ease or difficulty of participating in CBFM	3	1	Household
Benefits from Participation				
Amount of annual fodder harvest	Amount of fodder that household harvests from the forest	1	1	Household
Amount of annual fuelwood harvest	Amount of fuelwood that household harvests from forest	1	0	Household
Ecological benefits	Whether the household agree or not that forest species that is in their forest provide ecological benefits	3	0	Household
Economic benefits	Whether or not the household gains economic benefits from participating in CBFM	3	0	Community
Institutional benefits	whether or not the household gains institutional benefits from participating in CBFM	3	3	Community
Received aspired benefits	Whether the respondent thinks that he would receive aspired benefits after the final harvesting of trees or not;	1	1	Household
Received financial benefits	Whether or not the users received any financial benefit from forest	1	0	Household
Received training from forest department	Whether or not the respondent had received any trainings from forest department officials	4	4	Household
Social benefits	Whether or not user believe on local community society benefit from CFM	3	2	Community
Training participation	Whether or not participated in training provided by CBFM	7	4	Household
Bio-Physical				
Rice insufficiency period	Number of rice shortage months in a year	5	3	Household
Access to road	Household access and satisfaction of roads or not	1	0	Household
Annual precipitation level	Average annual precipitation (mm)	1	1	Community
Distance to all season road	Distance from household to all season road in KM	6	2	Household
Distance to forest	Distance between residence and community forests	35	16	Household
Distance to market	Distance between residence and closest market	21	7	Household
Farm size	Household farm size	4	1	Household
Fodder sources	Household fodder sources	1	1	Household
Forest area allotted per household	Community forest area allotted per household	1	0	Community

Forest condition	Condition of the community forest	9	5	Community
Forest degraded index	Forest degraded index	1	1	Community
Forest product collection	Whether or not household collect forest products	6	2	Household
Forest product exploitation type	Type of forest product households extracts or uses from the forests e	1	0	Household
Forest productivity improvement	Whether or not CBFM program is improving forest productivity	1	0	Community
Forest size	Total forest area under CBFM	20	8	Community
Fuelwood sources	Households fuelwood sources like forest, private land etc.	3	2	Household
Fuelwood use	Average amount of fuelwood uses by household	2	0	Household
Household elevation	The elevation of household location	1	1	Household
Household land ownership within PA	If household has land on protected are or not	1	1	Community
Household location	Location of the respondent household	1	1	Household
Land productivity	Land productivity per hectare	1	1	Household
Private woodlot ownership	Household/user have ownership of private woodlot or not	3	2	Household
Residence in high mountainous area	Household residence in high or low mountainous areas	3	3	Household
State forested land occupancy	Percent of land in the state that is forested	1	1	Household
Status of natural capital	Natural satisfaction within community across class and other bridging variables	1	1	Community
Timber received	Annual timber received by the household	1	1	Household
Time to reach forest	Time to reach forest from household	4	1	Household
Water availability	Number of months household have water availability for agriculture	2	1	Household
Economic				
NTFP Income	Household share of NTFPs income	8	3	Household
Other benefits from forest	Whether or not household have received other than monitory benefits from forest	1	1	Household
PES benefits	Whether or not household received PES benefits	1	1	Household
Alternative sources of livelihood	Whether or not households have alternative sources of livelihood	1	1	Household
Annual crop harvests	Household annual crop harvests amount	2	1	Household
Annual income	Total annual household income	26	11	Household
Annual income from forest	Household annual income from forest	1	1	Household
Asset value	Household asset value	6	2	Household
Capital per capita	Household capital divided by household size	4	1	Household
Consumption per capita	Household consumption divided by household size	4	1	Household
Credit access	Whether or not household have credit access for purchasing inputs	1	0	Household
Credit received	Whether or not household have received credits from CBFM	2	2	Household
Crop land ownership	Household have own crop land or not	1	1	Household
Duration of food insecurity	Duration of food insecurity that households have to endure	2	1	Household

Economic benefits from forest	Whether or not households received economic benefits from forest	1	1	Household
Employment status	Whether or not household head is employment	1	1	Household
Extension services	Whether or not household receives extension services	1	0	Household
Family members in occupation	The number of family members engaged in occupation	4	4	Household
Fodder received	Annual amount of fodder received from CBFM	1	0	Household
Food shortage duration	Months of food shortage of the household	3	3	Household
Forest benefit status	Whether or not household derived benefits from forest	1	1	Household
Forest benefits expectation	Whether or not household expect benefits from forest	4	0	Household
Fraction of income derived from forests	Percent of forest income share to total households income	6	6	Household
Fuelwood received	Annual amount of fuelwood received from CBFM	1	0	Household
Government expenses to education	Percent of government expenditures on education	1	1	Household
Household expenditure	Total gross annual expenditure of household	7	4	Household
Household income sources	Sources from where households income depends	3	0	Household
Household land renting	Whether or not household rent land	1	0	Household
Income before CBFM	Household income before project (amount)	1	1	Household
Income from cash crop	Whether or not household have annual income from cash crop	1	1	Household
Income from charcoal	Whether or not household have annual income from charcoal	1	1	Household
Income from fish	Whether or not household have annual income from fish farming	1	1	Household
Income inequality index	Household income inequality index	1	1	Household
Indirect use of forest	Whether or not households involve in indirect forest use	1	1	Household
Land inequality index	Households land inequality index	1	1	Household
Land ownership	Respondent have land ownership (land title) or not	6	3	Household
Land rented size	Whether or not household rented land	1	0	Household
Land size	Total land owned by the households	50	22	Household
Land tenure type	Inherited land tenure versus other means of acquiring land	5	4	Household
Level of perceived benefits	Household level of perceived benefits (high, low, or none)	1	1	Household
Livestock income	Percent of annual livestock income share to total household income	1	1	Household
Livestock unit	Total number of livestock’s owned by household	20	8	Household
Loss from CF program	Whether or not CF program bring households losses	1	0	Household
Monetary benefit from CF	Whether or not household have received monetary benefit from CF	1	0	Household
Monthly income	Households monthly income	3	2	Household
Natural resource dependency for income	Whether or not household depends on natural resource dependency for income	1	0	Household
Non-farm income share	Percent of non-farm income share	1	1	Household

Non-forested income	Increasing non-forested income from CBFM or not	1	1	Household
Number of extension calls	Number of extension calls to the households	1	0	Household
Number of family labor	Total number of family labor in the household	5	2	Household
Off farm employment	Whether or not household head have off farm employment	1	1	Household
Off farm income	Whether or not household have off farm income	7	6	Household
Past economic capital	Household level of past economic capital	1	0	Household
Payment for forest plot	Amount paid by household for forest plot	4	2	Household
Private woodlot ownership	Household/user have ownership of private woodlot or not	2	1	Household
Remittance received	Whether or not household received remittances in the past year	2	0	Household
Share of PES income	Percent share of PES income out of total household income	2	1	Household
Governance				
FUG meetings attended	Number of FUG meetings organized annually	1	1	Community
	Total number of FUG meetings attended within 90 days	1	0	Household
Forest officers visits to households	Whether or not forest officers visit to household to encourage them for participation and meetings	1	1	Household
Received benefit	Whether or not household/user received any benefits from CBFM	1	0	Household
Acceptance of participatory forestry	Whether local people accepted participatory forestry gladly or not	1	1	Community
Access to forest credit services	Household access and satisfaction of credit or not	2	1	Household
Access to forest products	Household satisfaction with access to forest products or not	3	2	Household
Access to fuel for cold season	Whether or not CBFM guarantee fuel for cold season	1	1	Household
Access to market information	Household access to market information or not	1	1	Household
Access to nutrition	Household access to viable food or not	1	1	Household
Advice provision	Whether or not users have received advice from forest office	4	3	Household
Agree with benefit sharing	Respondent agree with CBFM benefit sharing or not	1	1	Household
Agreement status	Whether or not the contractual agreement period was completed	1	1	Household
Attitude towards sustainable forest management	Favorable attitude or not towards sustainable management	2	2	Household
Awareness and training	Whether or not household received awareness and training	1	1	Household
Awareness on deforestation	Whether or not household received awareness of deforestation	5	3	Household
Awareness on forest policy	Whether or not household received awareness on policy or not deforestation	2	2	Household
Clear objective of CBFM	Clarity of households/users on forest goals and objectives or not	1	1	Household
Cohesiveness towards CF	Households level of cohesiveness towards CBFM	1	0	Household
Community awareness level	Community awareness level on forests	1	1	Community

Conflict mitigation strategy	Community forest program could mitigate conflict between forest authority and user or not	1	1	Community
Conflicts with forest department	Whether the users had conflicts with the forest department officials or not	1	0	Community
Connection with information channels and resources	Whether or not household is connected with information channels and resources	1	1	Household
Contributed to tree farming fund	Whether the respondent had been contributing to Tree Farming Funds or not	1	1	Household
Cooperation from forest authority	Whether forest authority cooperates or not	4	4	Household
Decision process	Whether the decision process adopted to participate in CBFM is fair or not	3	0	Household
Disrupted interest of users	Whether or not CF practice disrupted users interest	1	1	Household
Duration of forest membership	Number of years since household is a member of CBFM	3	2	Household
Effectiveness of management plan	Perception of effectiveness of CBFM plan	3	3	Community
Equitable benefit sharing	Whether or not CBFM practice equitable benefit sharing	1	1	Community
Executive committee member	Whether or not household have served as executive committee member	4	3	
Extension of forest conservation methods	Whether or not CBFM does extension of forest conservation methods	1	1	Community
Forest committee accountability	Whether or not forest committee have lack of accountability	1	0	Community
Forest management approached adopted	Forest management approach adopted by the CBFM	1	1	Community
Forest management structure adopted	Forest management structure adopted by the CBFM	2	2	Community
Forest users visit to forest office	Number of times that forest users visits to forest offices annually	1	1	Household
Fulfilment of agreements by forest office	If the household feels agreement has been upheld by forest office or not	2	2	Household
Governance structure	Household satisfaction of governance structures or not	3	0	Household
Household visits to forest office	Annual total number of visits to forest/project management office	1	1	
Implementation of conservation policies and programs	Implementing conservation policies and programs	1	0	Community
Implementation of deforestation program	CBFM implementing deforestation program or not	1	0	Community
Improving knowledge and awareness	Whether or not CBFM program is improving users knowledge and awareness	1	1	Household
Information received on CBFM	Whether or not household received information on CBFM	3	2	Household
Information sharing provision	Whether or not CBFM have information sharing provisions	2	2	Community
Institution regime type	Type of CBFM regime adopted (joint forest management or community based forest management)	3	2	Community
Interactions with forest officials	Whether or not households interacts with forest officials	2	2	Household

Knowledge on CBFM	Whether or not household have knowledge on CBFM	3	2	Household
Knowledge on forest conservation	Whether or not household have knowledge on forest conservation	2	2	Household
Lack of sense of ownership	Users have lack of sense of ownership towards forests or not	1	1	Household
Leadership quality	CBFM Leadership quality perceived by the households	4	1	Household
Leadership status	Respondent leadership status in society	1	2	Household
Leadership style	Type of CBFM leadership style (manipulative, authoritarian, participative and charismatic)	7	6	Household
Local forest institutions	Number of forest institutions in area	1	1	Community
Meetings held	Number of meetings held by CFUG last year	4	2	Community
Monitoring of forest plan implementation	Whether or not CBFM monitors implementation of forest plans	1	0	Household
Participatory boundary delineation	Whether or not CBFM specifying boundaries with people participation	1	1	Household
Policy awareness	Self-reported awareness of any forestry laws/ policies backing participation of local communities in forest resources management or not	4	0	Household
Property rights assignment	Whether or not well defined and enforced property rights over the forest assigned to the users	1	1	Household
Record keeping	Whether or not CBFM keeps working record	1	0	Household
Satisfaction on forest product regulation rules	Whether or not users are satisfied with the regulation of forest product extraction	1	1	Household
Satisfaction on participation rules	Whether or not users have satisfaction with participatory forestry rules	1	0	Household
Satisfied with species planted	Whether or not user have satisfaction with species planted or not	1	1	Household
Secure land rights	Household having secure land rights or not	3	2	Household
Sustainable and long-term planning	Whether or not CBFM have sustainable and long-term planning for forest productivity	1	1	Community
Technical assistance received	Whether or not household have received technical assistance	4	3	Household
Training opportunity	Whether or not CBFM provides training opportunity to household	2	2	Household
Institutional arrangement				
Forest access rights	Whether or not household have rights of access to forest	4	1	Household
Investment in local infrastructure	Whether or not CBFM invests in local community infrastructure development	1	0	Community
Involvement in executive committee	Whether or not household have previous involvement in forest management committee/EC	6	3	Household
Membership of farmer group	Whether or not household is a member of farmer group	4	1	Household
Member of local group	Whether or not household is a member of local groups	3	3	Household
Membership of forest association	Whether or not household is a member of community based forest association	3	1	Household
Participation in training	Whether or not household have participated in training provided by CBFM	2	12	Household
Received capacity building trainings	Whether or not users have received capacity building training	1	1	Household

Received extension services	Whether or not the users have received extension services	12	5	Household
Social				
Attachment to community	Whether or not household have attachment to the community	3	3	Household
Benefit received from forest	Whether or not household received benefits from forest	4	2	Household
Caste	Caste of household head/respondent	24	12	Household
Children in household	Number of children in household	3	0	Household
Communities number taking participation in CBFM	Number of communities per million people that took part of the program	1	1	Community
Community pressure	Whether or not households receives community pressure for participation or not	2	1	Household
Cultural protection	Whether or not community forests influence local culture	1	0	Household
Developing appropriate culture	Whether or not community forests develops appropriate culture	1	0	Community
Duration of living	How long household have been living in the community	2	1	Household
Elite domination	Perception on elite domination in decision making process	1	1	Household
Ethnic diversity	Ethnic diversity index of the community	1	0	Community
Family age	Average age in the family	3	1	Household
Females in household	Total female member in the household	3	1	Household
Forest dependency reduced	Whether or not CBFM is reducing livelihood dependence on forests	1	1	Household
Forest dependency for forest products	Whether or not household depends on forest for forest products	4	1	Household
Forest dependency for livelihood	Whether or not household depends on forest for livelihood	4	0	Household
Forest dependency ratio	Total use of forest goods, like fuelwood, fodder, timber, divided by total need per family	3	3	Household
Forest ecosystem knowledge	Whether or not household know the importance of forest ecosystem	1	1	Household
Forest resource self-sufficiency	Number of months household is self-sufficient to produce forest products like fodder and fuelwood	1	0	Household
Forest social values	Whether or not forest reinforce the social values	1	0	Household
Gender of respondent	Gender of respondent	65	29	Household
Gender proportion	Gender proportion in household	1	0	Household
Gender proportion in household	Gender proportion in household	4	1	Household
Household head age	Age of household head	32	8	Household
Household head education	Average years of schooling of the household head	20	3	Household
Household head gender	Household head gender	13	5	Household
Household highest level of education	Highest level of education received by the household	3	1	Household
Household size	Total family members in the household	58	17	Household
Households dependents number	Total number of dependents in the household	1	0	Household
Internal migrants	Total number of internal migrants in the household	2	2	Household

International migrants	Total number of international migrants in the household	2	0	Household
Lack of incentives	Whether or not CBFM have lack of incentives	1	1	Household
Level of awareness	Household level of awareness on CBFM	1	0	Household
Level of social security and cohesion	Level of social security and cohesion through local institution building	1	1	Community
Marital status of household head	Marital status of household head	9	4	Household
Marital status of respondent	Marital status of respondent	4	2	Household
Membership duration	Household membership duration in CBFM	2	2	Household
Migration status	Whether or not household member have migrated to other country for jobs/education	5	1	Household
Motivating and encouraging towards SFM	Whether or not CBFM motivates and encourage users towards sustainable forest management activities	1	0	Household
Occupation as farmer	Occupation of household head as farmer or not	3	1	Household
Occupation of household head	Household major occupation	9	4	Household
Occupation of respondent	Main occupation of respondent	3	2	Household
Organizational affiliation	Number of organizational affiliation of the household	4	2	Household
Perception in change of forest cover	Household perception in change of forest cover over last five years (increasing/decreasing)	2	1	Household
Perception on community forest	Users perception on community forestry	1	1	Household
Perception on forest management policy	Users perception on forest management policy	1	0	Household
Perception on forest species	Users perception on distribution and abundance of community forest species	3	2	Household
Political affiliation	The way the state voted in the presidential election (Republican or Democratic)	1	1	Household
Purpose of CBFM implementation	Whether or not user/household knows the purpose of implementing CBFM program	1	0	Household
Regional provenance	If the household is native or not	3	3	Household
Religion of household	Religion practiced by household	7	3	Household
Residence status	Household residence is indigenous/ native or migrant	7	0	Household
Resource support	Level of resource support from forest during forest development phase	4	4	Household
Respondent age	Age of respondent	29	13	Household
Respondent education	Education level of respondent	60	22	Household
Restriction on charcoal and timber production	Whether or not CBFM have restriction on charcoal and timber harvesting	1	2	Household
Satisfaction on CBFM program	Whether or not users have satisfaction with CBFM program	3	1	Household
Self-efficacy to participate	Recognition of own ability to participate in CBFM	3	3	Household
Shocks	Value of 3 main shocks suffered by household over the past year	6	0	Household
Social capital	Social satisfaction within community across class and other bridging variables	2	1	Household
Social cohesiveness	Level of social cohesiveness	4	4	Community

Social groups	Total number of social groups to which the household is attached	1	1	Household
Time to sell forest products	Time taken in hours to sell forest products	1	0	Household
Tribal cohesion	Whether or not household belongs to any main tribal in the country	2	0	Household
User group size	Number of users associated with CFUG	1	1	Community
Wellbeing class	Household wellbeing class (Rich to pro-poor)	13	5	Household
Willingness to pay	Household willingness to pay for forest	4	2	Household
Working population	Percent of the state population between 18-65	1	1	Household
Years of membership	Years of CF membership of the household	2	1	Household
Years of residence	Total years of residence of household in that place	5	4	Household

***Household level variables** are specific to the respondents' individual households. They include data that relates to the household's characteristics, behaviors, and experiences. **Community-level variables** are broader and relate to the community as a whole. They reflect the community's collective characteristics, governance, infrastructure, and policies.

Note: The terms 'users,' 'respondents,' or 'farmers' are used interchangeably across different studies, but all refer to the survey respondents who are participating in CBFM.

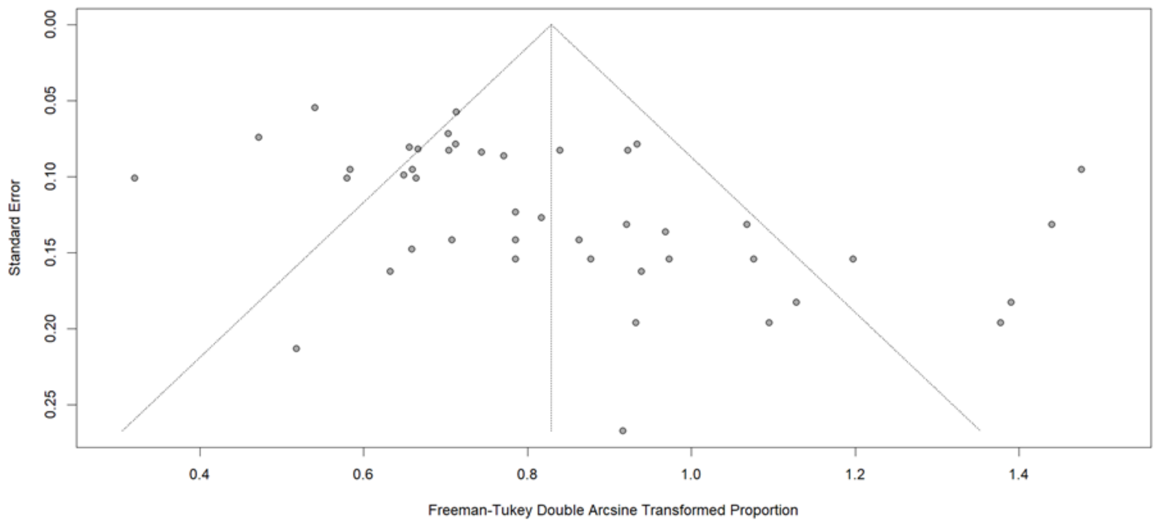

Fig. S3: A funnel plot shows the variability (standard error) between different study cases against the pooled proportion. Each dot represents a study. The x-axis (Freeman-Tukey Double Arcsine Transformed Proportion) shows the prevalence proportion from each study after the Freeman-Tukey transformation. The Freeman-Tukey transformation stabilizes the proportion variance. The y-axis, which represents standard error, displays the corresponding standard error for each study. Smaller studies having small sample sizes (with larger standard errors) will appear lower on the plot, while larger studies having larger sample sizes (with smaller standard errors) will appear higher. The symmetric distribution of studies above and below the pooled proportion indicates that there is no study bias.

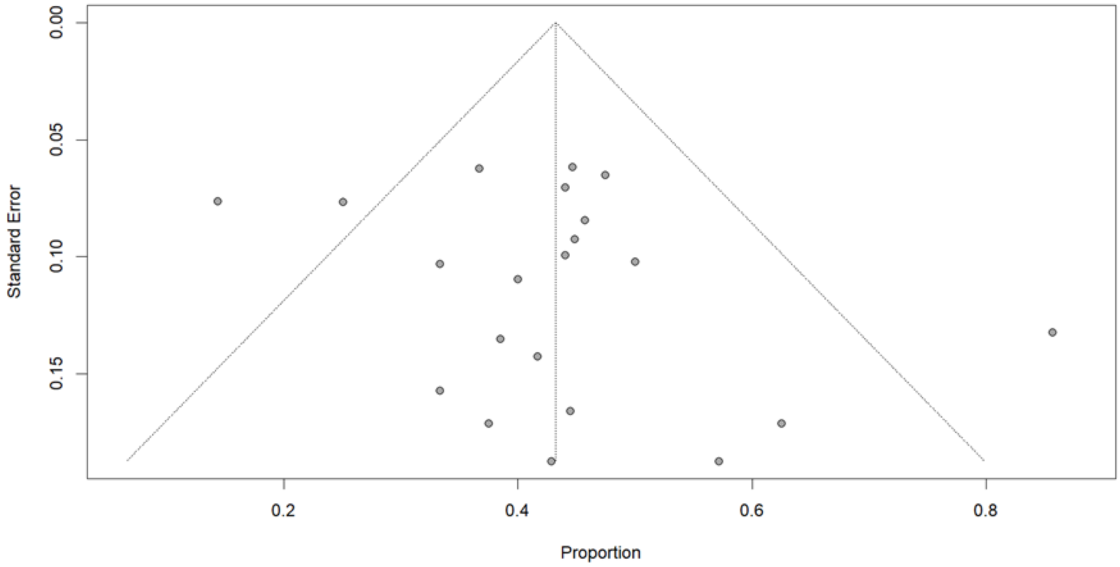

Fig. S4: A funnel plot shows the variability (standard error) between different consistently tested predictors against the pooled proportion. Each dot represents a predictor. Here, the symmetric distribution of studies above and below the pooled proportion indicates no study bias. The high standard error means a small sample size, and vice versa.

Table S3: List of studies included in meta-analysis

1. Adams, oduro, & Ansong. (2017). Factors Affecting Stakeholders' Participation in Collaborative Forest Management: The Case of Krokosua Hills Forest Reserve in Ghana. *Journal of Energy and Natural Resource Management*, *3*(2), 66–73.
2. Adhikari, S., Kingi, T., & Ganesh, S. (2014, July). Incentives for community participation in the governance and management of common property resources: the case of community forest management in Nepal. *Forest Policy and Economics*, *44*, 1–9. https://doi.org/10.1016/j.forpol.2014.04.003
3. Agrawal, A., & Gupta, K. (2005, July). Decentralization and Participation: The Governance of Common Pool Resources in Nepal's Terai. *World Development*, *33*(7), 1101–1114. https://doi.org/10.1016/j.worlddev.2005.04.009
4. Akamani, K., & Hall, T. E. (2015, January). Determinants of the process and outcomes of household participation in collaborative forest management in Ghana: A quantitative test of a community resilience model. *Journal of Environmental Management*, *147*, 1–11. https://doi.org/10.1016/j.jenvman.2014.09.007
5. Apipoonyanon, C., Kuwornu, J. K. M., Szabo, S., & Shrestha, R. P. (2019, June 24). Factors influencing household participation in community forest management: evidence from Udon Thani Province, Thailand. *Journal of Sustainable Forestry*, *39*(2), 184–206. https://doi.org/10.1080/10549811.2019.1632211
6. Bakala, F., Asfaw, M., & Tadesse, B. (2021, January 7). Factors Influencing Household Participation in a Participatory Forest Management Scheme: Bench-Sheko Zone, Southwest Ethiopia. *Journal of Sustainable Forestry*, *41*(10), 909–921. https://doi.org/10.1080/10549811.2020.1867184
7. Basu, J. P. (2021, March 1). Forest participation of local communities: a study of a tribal dominated region in India. *Journal of Social and Economic Development*, *23*(1), 180–201. https://doi.org/10.1007/s40847-020-00142-w
8. Bista, R., Graybill, S., Zhang, Q., Bilsborrow, R. E., & Song, C. (2023, January 24). Influence of Rural Out-Migration on Household Participation in Community Forest Management? Evidence from the Middle Hills of Nepal. *Sustainability*, *15*(3), 2185. https://doi.org/10.3390/su15032185
9. Brännlund, R., Sidibe, A., & Gong, P. (2009, November). Participation to forest conservation in National Kabore Tambi Park in Southern Burkina Faso. *Forest Policy and Economics*, *11*(7), 468–474. https://doi.org/10.1016/j.forpol.2009.05.005
10. Chhetri, B. B. K., Johnsen, F. H., Konoshima, M., & Yoshimoto, A. (2013, May). Community forestry in the hills of Nepal: Determinants of user participation in forest management. *Forest Policy and Economics*, *30*, 6–13. https://doi.org/10.1016/j.forpol.2013.01.010
11. Coulibaly-Lingani, P., Savadogo, P., Tigabu, M., & Oden, P. C. (2011, April). Factors influencing people's participation in the forest management program in Burkina Faso, West Africa. *Forest Policy and Economics*, *13*(4), 292–302. https://doi.org/10.1016/j.forpol.2011.02.005

12. Derkyi, M. A. A., Appau, Y., & Boakye Boadu, K. (2021, November 2). Factors influencing community participation in the implementation and monitoring of FLEGT-VPA in Ghana. *Forestry Economics Review*, *3*(1), 19–37. https://doi.org/10.1108/fer-07-2020-0010
13. Dolisca, F., Carter, D. R., McDaniel, J. M., Shannon, D. A., & Jolly, C. M. (2006, December). Factors influencing farmers' participation in forestry management programs: A case study from Haiti. *Forest Ecology and Management*, *236*(2–3), 324–331. https://doi.org/10.1016/j.foreco.2006.09.017
14. Ekanayake, E. M. B. P., Xie, Y., & Ahmad, S. (2021, August 7). Rural Residents' Participation Intention in Community Forestry-Challenge and Prospect of Community Forestry in Sri Lanka. *Forests*, *12*(8), 1050. https://doi.org/10.3390/f12081050
15. Farouque, M. G., Fuyuki, K., & Takashino, N. (2017). Attitudesof local people towards community-based forest management: Astudy of a Sal forest area in Bangladesh. *International Journal of Agricultural Extension and Rural Development* , *4*(1), 263–273.
16. Gashu, K., & Aminu, O. (2019, January 20). Participatory forest management and smallholder farmers' livelihoods improvement nexus in Northwest Ethiopia. *Journal of Sustainable Forestry*, *38*(5), 413–426. https://doi.org/10.1080/10549811.2019.1569535
17. Jana, S. K., Lise, W., & Ahmed, M. (2014, December). Factors affecting participation in joint forest management in the West Bengal state of India. *Journal of Forest Economics*, *20*(4), 317–332. https://doi.org/10.1016/j.jfe.2014.09.003
18. Janata, & paulos. (2017). Farmer's Participation in Participatory Forest Management and Factors Affecting its Performance (The Case of Sodo Zuriya District, Wolaita Zone , Ethiopia) . *Journal of Economics and Sustainable Development* , *8*(9), Article ISSN 2222-1700.
19. Jumbe, C. B., & Angelsen, A. (2007, May). Forest dependence and participation in CPR management: Empirical evidence from forest co-management in Malawi. *Ecological Economics*, *62*(3–4), 661–672. https://doi.org/10.1016/j.ecolecon.2006.08.008
20. Kazungu, M., Zhunusova, E., Kabwe, G., & Günter, S. (2021, March 3). Household-Level Determinants of Participation in Forest Support Programmes in the Miombo Landscapes, Zambia. *Sustainability*, *13*(5), 2713. https://doi.org/10.3390/su13052713
21. Kerse. B. (2016). Factors Affecting Local People Participation in Forest Managed for Carbon Sequestration: The Case of Mount Damota, Southern Ethiopia. *Developing Country Studies* , *6*(5), Article ISSN 2224-607X (Paper) ISSN 2225-0565 (Online).
22. Kimengsi, J. N., & Deodatus Ngu, N. (2022, September). Community participation and forest management dynamics in rural Cameroon. *World Development Perspectives*, *27*, 100442. https://doi.org/10.1016/j.wdp.2022.100442
23. Lestari, S., Kotani, K., & Kakinaka, M. (2015, March). Enhancing voluntary participation in community collaborative forest management: A case of Central Java, Indonesia. *Journal of Environmental Management*, *150*, 299–309. https://doi.org/10.1016/j.jenvman.2014.10.009
24. Lise, W. (2000, September). Factors influencing people's participation in forest management in India. *Ecological Economics*, *34*(3), 379–392. https://doi.org/10.1016/s0921-8009(00)00182-8

25. Luswaga, H., & Nuppenau, E. A. (2020, January 27). Participatory Forest Management in West Usambara Tanzania: What Is the Community Perception on Success? *Sustainability*, *12*(3), 921. https://doi.org/10.3390/su12030921
26. Maskey, V., Gebremedhin, T. G., & Dalton, T. J. (2006, January). Social and cultural determinants of collective management of community forest in Nepal. *Journal of Forest Economics*, *11*(4), 261–274. https://doi.org/10.1016/j.jfe.2005.10.004
27. Mbeche, R., Ateka, J., Herrmann, R., & Grote, U. (2021, August). Understanding forest users' participation in participatory forest management (PFM): Insights from Mt. Elgon forest ecosystem, Kenya. *Forest Policy and Economics*, *129*, 102507. https://doi.org/10.1016/j.forpol.2021.102507
28. Mohammed, J., Osei-Fosu, A. K., & Yusif, H. (2017, December 1). Factors influencing households' participation in forest management in the northern region of Ghana. *Independent Journal of Management & Production*, *8*(4), 1324. https://doi.org/10.14807/ijmp.v8i4.631
29. Musyoki, J. K., Mugwe, J., Mutundu, K., & Muchiri, M. (2013, January 10). Determinants of Household Decision to Join Community Forest Associations: A Case Study of Kenya. *ISRN Forestry*, *2013*, 1–10. https://doi.org/10.1155/2013/902325
30. Musyoki, J. K., Mugwe, J., Mutundu, K., & Muchiri, M. (2016, March 3). Factors influencing level of participation of community forest associations in management forests in Kenya. *Journal of Sustainable Forestry*, *35*(3), 205–216. https://doi.org/10.1080/10549811.2016.1142454
31. Negi, S., Pham, T., Karky, B., & Garcia, C. (2018, March 13). Role of Community and User Attributes in Collective Action: Case Study of Community-Based Forest Management in Nepal. *Forests*, *9*(3), 136. https://doi.org/10.3390/f9030136
32. Okumu, B., & Muchapondwa, E. (2020, April). Determinants of successful collective management of forest resources: Evidence from Kenyan Community Forest Associations. *Forest Policy and Economics*, *113*, 102122. https://doi.org/10.1016/j.forpol.2020.102122
33. Oli, B., & Treue, T. (2015, September 3). Determinants of participation in Community Forestry in Nepal. *International Forestry Review*, *17*(3), 311–325. https://doi.org/10.1505/146554815815982693
34. Paudyal, R., Thapa, B., Neupane, S., & KC, B. (2018, September 29). Factors Associated with Conservation Participation by Local Communities in Gaurishankar Conservation Area Project, Nepal. *Sustainability*, *10*(10), 3488. https://doi.org/10.3390/su10103488
35. Poungngamchuen, J., & Namvises, K. (2012). Peopleûs Participation in Dong Na Tham CommunityForest Management Project, Ubon Ratchathani, Thailand. *Kasetsart J.*, *33*, 486–498.
36. Ranjit, Y. (2016, February 8). Determinants of People's Participation in Forest Protection and Management: A Study in Kaski, Nepal. *Economic Journal of Development Issues*, 175–186. https://doi.org/10.3126/ejdi.v17i1-2.14527
37. Salam, M., Noguchi, T., & Koike, M. (2005, January). Factors influencing the sustained participation of farmers in participatory forestry: a case study in central Sal forests in Bangladesh. *Journal of Environmental Management*, *74*(1), 43–51. https://doi.org/10.1016/j.jenvman.2004.08.007

38. Savari, M., Eskandari Damaneh, H., & Eskandari Damaneh, H. (2020, June 20). Factors influencing local people's participation in sustainable forest management. *Arabian Journal of Geosciences*, *13*(13). https://doi.org/10.1007/s12517-020-05519-z
39. Sinha, H., & Suar, D. (2005, June). Leadership and people's participation in community forestry. *International Journal of Rural Management*, *1*(1), 125–143. https://doi.org/10.1177/097306800400100107
40. Soe, K. T., & Yeo-Chang, Y. (2019, March). Perceptions of forest-dependent communities toward participation in forest conservation: A case study in Bago Yoma, South-Central Myanmar. *Forest Policy and Economics*, *100*, 129–141. https://doi.org/10.1016/j.forpol.2018.11.009
41. Subedi, M. R., & Timilsina, Y. P. (2016, January 2). Evidence of User Participation in Community Forest Management in the Mid-hills of Nepal: A Case of Rule Making and Implementation. *Small-Scale Forestry*, *15*(2), 257–270. https://doi.org/10.1007/s11842-015-9321-y
42. Tadesse, S., Woldetsadik, M., & Senbeta, F. (2017, October 2). Forest users' level of participation in a participatory forest management program in southwestern Ethiopia. *Forest Science and Technology*, *13*(4), 164–173. https://doi.org/10.1080/21580103.2017.1387613
43. Wall, B., Straka, T., & Miller, S. (2006, September 1). An Econometric Study of the Factors Influencing Participation in Urban and Community Forestry Programs in the United States. *Arboriculture & Urban Forestry*, *32*(5), 221–228. https://doi.org/10.48044/jauf.2006.028
44. Walle, Y., & Nayak, D. (2022, June). Factors determining the participation of natural resource cooperative members in forest management: A study of dry forest area in Ethiopia. *Trees, Forests and People*, *8*, 100241. https://doi.org/10.1016/j.tfp.2022.100241
45. Waruingi, E., Mbeche, R., & Ateka, J. (2021, April). Determinants of forest dependent household's participation in payment for ecosystem services: Evidence from Plantation Establishment Livelihood Improvement Scheme (PELIS) in Kenya. *Global Ecology and Conservation*, *26*, e01514. https://doi.org/10.1016/j.gecco.2021.e01514
46. Wambugu, E.W., Obwoyere, G.O., & Kirui, B.K. (2017). Socioeconomic Factors that determine community participation in forest management and conservation of adjacent ecosystems: A case of Aberdare forest, Kenya. Journal of Ecology and the Natural Environment, 9(10), 165–176. https://doi.org/10.5897/JENE2017.0666
47. Zande, R., & Mzuza, M. K. (2022). An Investigation of the Factors Influencing Community Participation in Forest Management: A Case of Balaka District, Malawi. *Journal of Geoscience and Environment Protection*, *10*(03), 84–95. https://doi.org/10.4236/gep.2022.103007
